# Spotting Local Environments in Self-Assembled Monolayer-Protected Gold Nanoparticles

**DOI:** 10.1021/acsnano.2c08467

**Published:** 2022-12-02

**Authors:** Cristian Gabellini, Maria Şologan, Elena Pellizzoni, Domenico Marson, Mario Daka, Paola Franchi, Luca Bignardi, Stefano Franchi, Zbyšek Posel, Alessandro Baraldi, Paolo Pengo, Marco Lucarini, Lucia Pasquato, Paola Posocco

**Affiliations:** † Department of Engineering and Architecture, University of Trieste, 34127 Trieste, Italy; ‡ Department of Chemical and Pharmaceutical Sciences and INSTM Trieste Research Unit, University of Trieste, 34127 Trieste, Italy; § Department of Chemistry “G. Ciamician”, University of Bologna, I-40126 Bologna, Italy; ∥ Department of Physics, University of Trieste, 34127 Trieste, Italy; ⊥ Elettra Sincrotrone Trieste, 34149 Basovizza, Trieste, Italy; # Department of Informatics, Jan Evangelista Purkyně University, 400 96 Ústí nad Labem, Czech Republic

**Keywords:** mixed monolayers, fluorinated nanoparticles, ESR, multiscale modeling, machine learning, SOAP, nanoconfinement

## Abstract

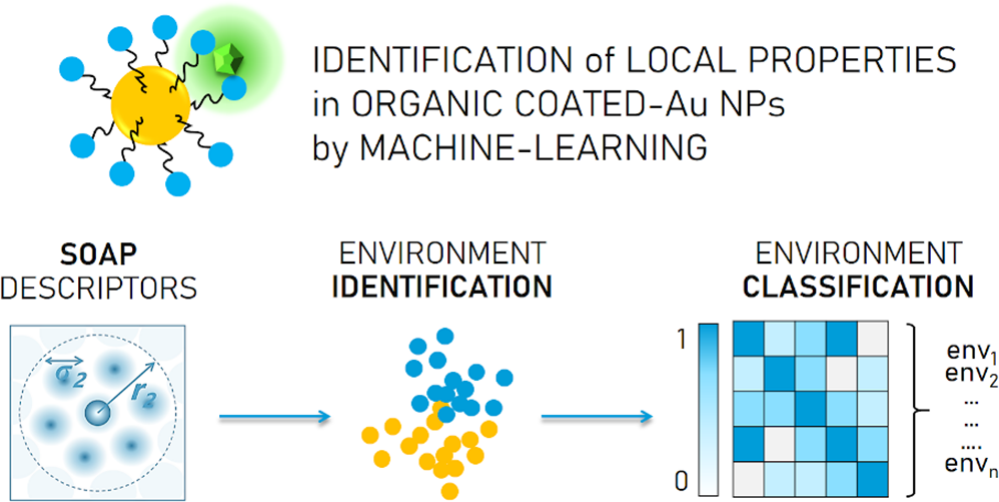

Organic–inorganic (O–I) nanomaterials are versatile platforms for an incredible high number of applications, ranging from heterogeneous catalysis to molecular sensing, cell targeting, imaging, and cancer diagnosis and therapy, just to name a few. Much of their potential stems from the unique control of organic environments around inorganic sites within a single O–I nanomaterial, which allows for new properties that were inaccessible using purely organic or inorganic materials. Structural and mechanistic characterization plays a key role in understanding and rationally designing such hybrid nanoconstructs. Here, we introduce a general methodology to identify and classify local (supra)molecular environments in an archetypal class of O–I nanomaterials, i.e., self-assembled monolayer-protected gold nanoparticles (SAM-AuNPs). By using an atomistic machine-learning guided workflow based on the Smooth Overlap of Atomic Positions (SOAP) descriptor, we analyze a collection of chemically different SAM-AuNPs and detect and compare local environments in a way that is agnostic and automated, i.e., with no need of *a priori* information and minimal user intervention. In addition, the computational results coupled with experimental electron spin resonance measurements prove that is possible to have more than one local environment inside SAMs, being the thickness of the organic shell and solvation primary factors in the determining number and nature of multiple coexisting environments. These indications are extended to complex mixed hydrophilic–hydrophobic SAMs. This work demonstrates that it is possible to spot and compare local molecular environments in SAM-AuNPs exploiting atomistic machine-learning approaches, establishes ground rules to control them, and holds the potential for the rational design of O–I nanomaterials instructed from data.

There is an intense interest in the rational design of organic–inorganic (O–I) hybrid nanomaterials.^1^ Installation of organic molecules and specifically thiol-containing ligands on a nanosized gold core is a primary example of such O–I nanoplatforms. Thanks to reproducible synthetic approaches that enable fine control over size, shape, surface chirality, and dispersion, the easiness to passivate the gold surface by the formation of a self-assembled monolayer (SAM) and to further introduce a variety of functional groups has enabled significant steps forward in the last years, granting access to a plethora of SAM-enabled gold nanoparticles (AuNPs) with functional properties.^2^ Indeed, the self-organization of ligands endows SAM-AuNPs with unique molecular recognition and sensing characteristics, which arise from the collective and cooperative behavior of the organic layer.^3,4^ The nanoconfinement imposed to surface-bound molecules dramatically influences their chemical and physical properties, as well as conformation.^5−8^ For instance, Kay studied the nanoparticle-confined hydrazone exchange.^9^ With the help of molecular dynamics calculations, the work demonstrated that at nanoscale SAM structure and conformational dynamics affects the transport properties and local concentration of reagent water involved in the exchange, the accessibility to the reaction sites, and ultimately the overall reaction kinetics. Grzybowski and collaborators conceived a mixed SAM-AuNP, in which longer ligands end in “gating units” able to control both the access and orientation of the incoming substrates with respect to the catalytic centers tethered at the end of shorter ligands. Gating, substrate- and site-selectivities derived from the molecular details of the on-particle molecular environment needed to be carefully designed.^10^ Mimicking the catalytic activity of proteins or their interaction with biological matter exploiting SAM-AuNPs has also been the object of growing exploration.^11−14^ The integration of bio-orthogonal catalytic systems such as transition-metal catalysts into nanoparticle scaffolds allowed the creation of synthetic catalytic nanosystems (nanozymes) able to replicate the complex behavior of natural enzymes in biological media.^15,16^ Hydrophobicity of surface motifs and monolayer compaction regulate the kinetic behavior of the nanozyme, together with temperature or pH.^17,18^

The examples cited above point out the beauty and complexity of surface confined environments in SAMs. They all rely on the local structure, dynamics, and solvation of the monolayer at atomic and nanoscale, although to a different extent. With a broad term, they exploit the features of *local (supra)molecular environments* in SAMs (Figure 1). For instance (Figure 1b), molecular structure, accessibility, surface morphology, and local reagent concentration change when the gate is open or closed. Thus, in this context, we can think of local (supra)molecular environments as regions of the monolayer with unique distinct fingerprints. The term encompasses multiple interconnected effects, such as atom density, ligand dynamics and conformation, monolayer structure, and ligand–ligand and ligand–solvent interactions as well as local solvation or substrate concentration (if any). As such, they are hard to anticipate and only few of them can be directly assessed with experiments by using techniques such as NMR,^19−21^ SANS,^22^ MALDI-TOF,^23^ and ESR;^24,25^ yet, these techniques suffer of some limitations, as the monolayer needs to be designed *ad hoc* for the specific technique.

**Figure 1:**
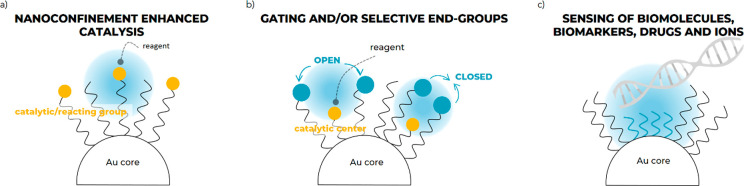
Exemplification of the concept of local (supra)molecular environment (highlighted in blue) in SAM-AuNPs and its exploitation. (a) If ligands contain a catalytic group and the surrounding molecules adopt specific cooperative conformation and order, 3D binding sites similar to those in enzymes may arise with enhanced catalytic properties. (b) The end group on the surface switches on/off the access to a catalytic center and grants selective diffusion to the organic layer, causing different local structural features and reagent concentration. (c) Heteroligand monolayers of two immiscible ligands lead to surface anisotropy with implications for surface related biological processes and sensing of biomolecules, biomarkers, and drugs.

Thus, we wondered if a general way to identify specific local settings in SAMs could exist. Molecular dynamics (MD) and coarse-grained simulations have been instrumental in retrieving information difficult to infer from experiments and in explaining the behavior of SAM-AuNPs at molecular and nanoscale with good reliability.^26−29^ Over recent years, the increasingly large amounts of data produced by these calculations have also been used by algorithms to extrapolate molecular patterns and predict (meta)stable configurations or structural motifs in complex matter.^30−32^

Here, in a proof-of-concept study, we introduce a two-step computational workflow able to detect first and then compare local (supra)molecular environments in SAM-AuNPs with no need of predefined information and minimal user intervention. It combines atomistic all-atom MD (AA-MD) calculations and the Smooth Overlap of Atomic Positions (SOAP) descriptors for machine-learning guided analysis. The retrieved local environments are then described and rationalized by MD calculations and supported by experiments of electron spin resonance (ESR), a spectroscopic technique highly sensitive to polarity changes in the local background perceived by a radical probe,^25^ that are carried out at different temperatures.

A set of AuNPs (roughly 4.0 nm in size), which support homo- and hetero (mixed)-SAMs composed of thiolates ending in positive (ligands **1** and **2**) or negative (ligands **3** and **4**) or zwitterionic (ligand **5**) charged end groups and short fluorinated ligands (ligand **6**) (Figure 2), is tested. We sought to augment the complexity of the monolayer by including fluorine containing mixed SAMs, which are particularly relevant for driving surface phase separation,^21,26^ controlling hydrophobicity or superphydrophobicity of surfaces,^33^ or tuning the molecule–NP interaction.^34^

**Figure 2:**
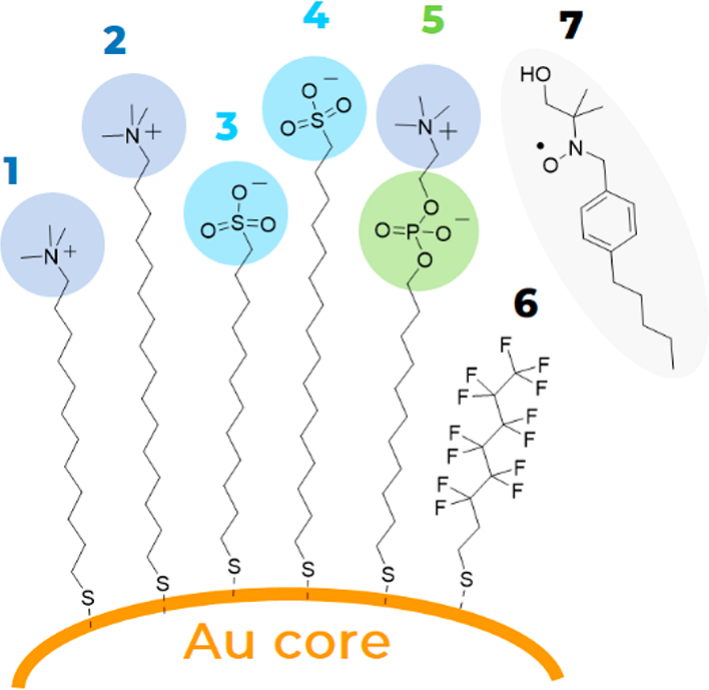
Structure of the thiolates **1**–**6** for the AuNP coating. Radical probe **7** for ESR investigation. Ligand **6** is used in mixed monolayers with **1**–**5**. Thiolates differ in nature and charge of the terminal group (**1** and **2**, a positively charged quaternary ammonium ion; **3** and **4**, a negatively charged sulfonate ion; **5**, a zwitterionic group, composed by a trimethylammonium and a phosphate group) as well as in length of the alkyl chain (C_12_ in **1**, **3**, **5**; C_16_ in **2**, **4**).

Hereafter, we adopt the following notation: **NP1** indicates a SAM of ligand **1** on AuNP while **NP1/6**, a SAM of ligand **1** and **6** on AuNP.

The paper is organized as follows: first, NP structure and properties from AA-MD simulations in solvent (water) are discussed; second, the computational approach for the identification and comparison of local motifs in different SAMs is illustrated and the outcomes considered; third, the results are interpreted in light of ESR investigation.

Overall, this work not only demonstrates that it is possible to spot local (supra)molecular environments in SAM-AuNPs by exploiting atomistic data-driven approaches but also is a step toward the design of functional nanoparticles with a programmable response.

## Results and Discussion

### MD-Derived SAM-AuNP Characterization

**Figure 3:**
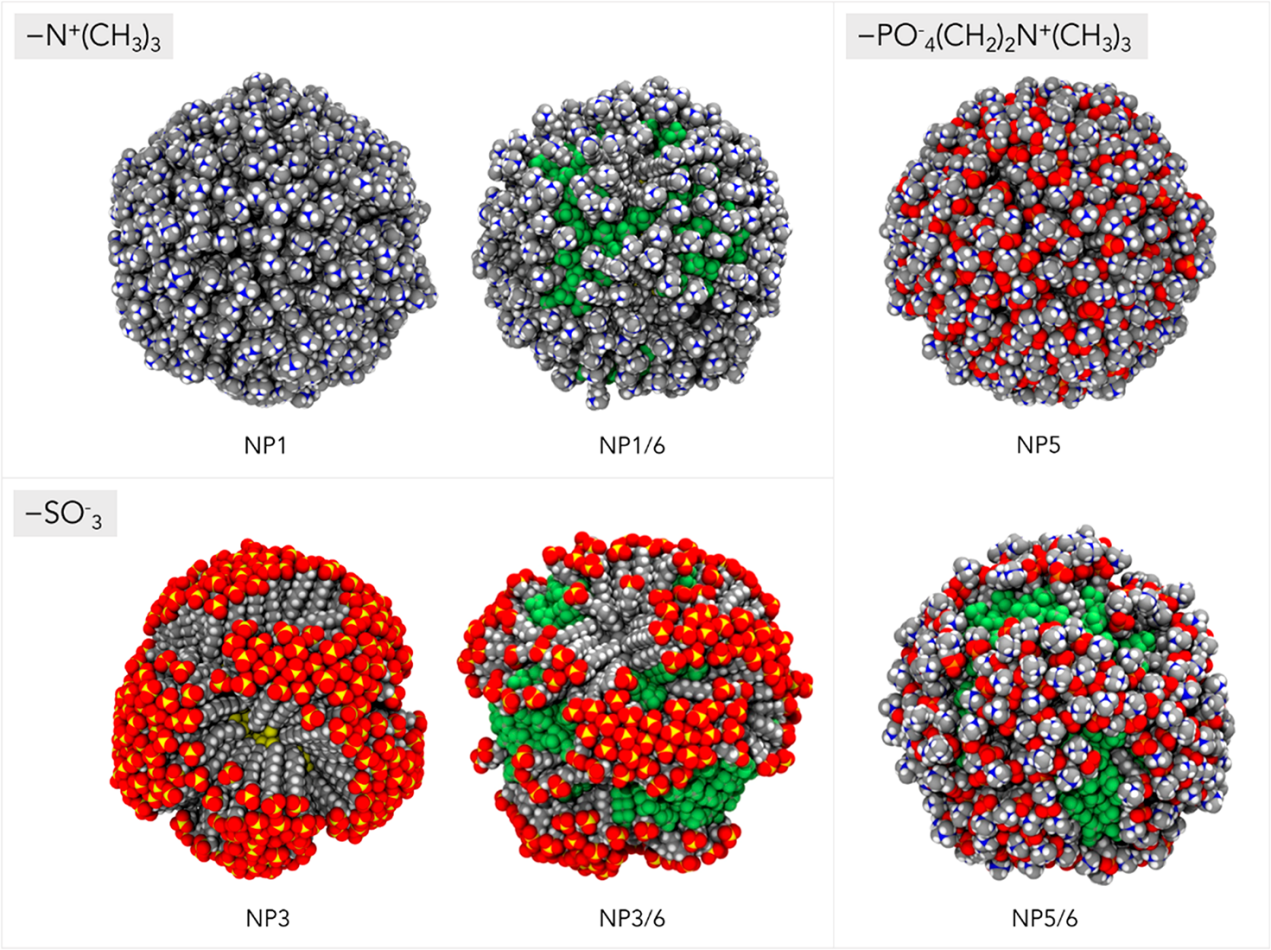
Representative molecular structures of homoligand **NP1**, **NP3**, and **NP5** AuNP and its heteroligand **NP1/6**, **NP3/6**, and **NP5/6** counterpart from molecular dynamics simulations in explicit solvent (water). For clarity, water and counterions are not shown. Color representation of atoms: C, gray; O, red; S, yellow; P, orange; N, blue; F, green; H, white.

The specific structure of the monolayer is imparted by the self-organization of the individual thiolates on the surface of the gold core. We have very recently demonstrated by calculations^28^ that the surface morphology depends on size and hydrogen bonding capability of the ligand end group, while other features, such as the alkyl chain length or the core size, affect the final ligand organization less. In particular, a large space-filling group like trimethylammonium or zwitterionic ones give rise to spatially uniform arrangements due to the steric hindrance of bulky terminal moieties; small end groups like sulfonate allow association of the chains in bundles, which instead leads to anisotropic shells (Figures 3 and S1). The combination of more than one kind of ligand in the shell has long been used in the nanoparticle community to tune nanoparticle solubility, wettability, interfacial properties, hydrophobic interactions for self-assembling nanoparticles, respond to the surrounding (bio)environment, and induce nanoscale surface morphologies.^4,35−39^

Indeed, when a mixture of dissimilar and/or immiscible molecules are employed to coat AuNPs, nanoscale domains may spontaneously form in the shell via ligand surface rearrangement.^40^ Fluorinated ligands are known to be highly lipophobic, and we have already tested their ability to trigger phase separation in 3D SAMs when used in combination with hydrogenated thiolates even at low molar fraction.^21,26^ Here, we have carried out auxiliary mesoscale simulations (to cope with the slow evolution of the phase separation at the nanoscale)^41^ coupled with AA-MD calculations in water to predict the pattern of organic shells containing fluorinated thiolates, namely, ligand **6**. For details on molecular models and simulations, see the Experimental Section and Supporting Information (SI) Section S3. Gold size, ligand density, and monolayer composition have been assigned to closely match those obtained experimentally (see SI Section S2).

The calculations confirm that, irrespective to the chemical nature and the chain length of the primary ligand (i.e., **1**–**5**), ligands **6** separate in small domains (Figures 3 and S1). For AuNPs bearing sulfonates and zwitterionic moieties as surface groups (namely, **NP3/6**, **NP4/6**, and **NP5/6**) these domains appear as elongated patches with an average width of 1.6–1.9 nm and length of 2.7–3.6 nm. Stripe-like patterns are indeed present on **NP1/6** and **NP2/6**, where the bulkier headgroups favor the formation of domain interfaces more (Figure S2).^42^

The phase separation does not alter the propensity of the most abundant ligand to associate in bundles. Thus, **NP3/6** and **NP4/6** have a spatially heterogeneous ligand distribution as is also observed for their homoligand counterpart **NP3** and **NP4** (Figures 3 and S1). The clustering (or bundling) of ligands can be quantified by means of Voronoi diagrams, which allow local density estimation through nearest neighbor analysis^43^ (Figures S3 and S4). In both monolayers, the presence of high-density regions where the chains form bundles is evident and these roughly correspond in number to those identified using a different clustering algorithm (e.g., HBDSCAN, see SI Table S1), supporting the presence of time-persistent aggregation of ligands. Regular and more uniform patterns instead characterize the Voronoi diagram of **NP1**, **NP3**, and **NP5** and their heteroligand partners **NP1/6**, **NP3/6**, and **NP5/6** (Figures S3 and S4) consistently with an isotropic distribution of the ligands around the gold core. The results also highlight that long ligands (i.e., HS-C_16_–FG) on nanoparticled induce more heterogeneous ligand distributions, which appeared clearly from the visual inspection of the diagrams (e.g., compare Figures S3a and S4a or Figures S3c and S4c). Yet, a simple measure is provided by the area dispersion index (ADI), which describes the spread of the tessellation cell areas (see SI Section S3.3 for how ADI is calculated) (Table S1). For **NP1**, ADI is equal to 2.24 and increases to 2.52 for **NP2**, indicating a broader distribution of the area available for each ligand; the increased local heterogeneity in long chains has also been seen by others,^44^ and it is promoted by higher interchain van der Waals interactions and higher free chain volume due to the increased radiality. For anisotropic shells like **NP3** and **NP4**, this phenomenon is less evident from ADI analysis (ADI is equal to 2.96 and 3.12, respectively) but still detectable in the diagrams. Adding a second ligand in the monolayer does not affect the overall monolayer structure yet impacts the ligand local order. Indeed, for almost all the heteroligand monolayers, the ADI decreases compared to the homoligand AuNP, thus indicating a more uniform distribution of the space available for each chain, likely because of the bulky fluorinated alkyl thiolates. Further structural analysis of the monolayer is available in Tables S1 and S2.

**Figure 4:**
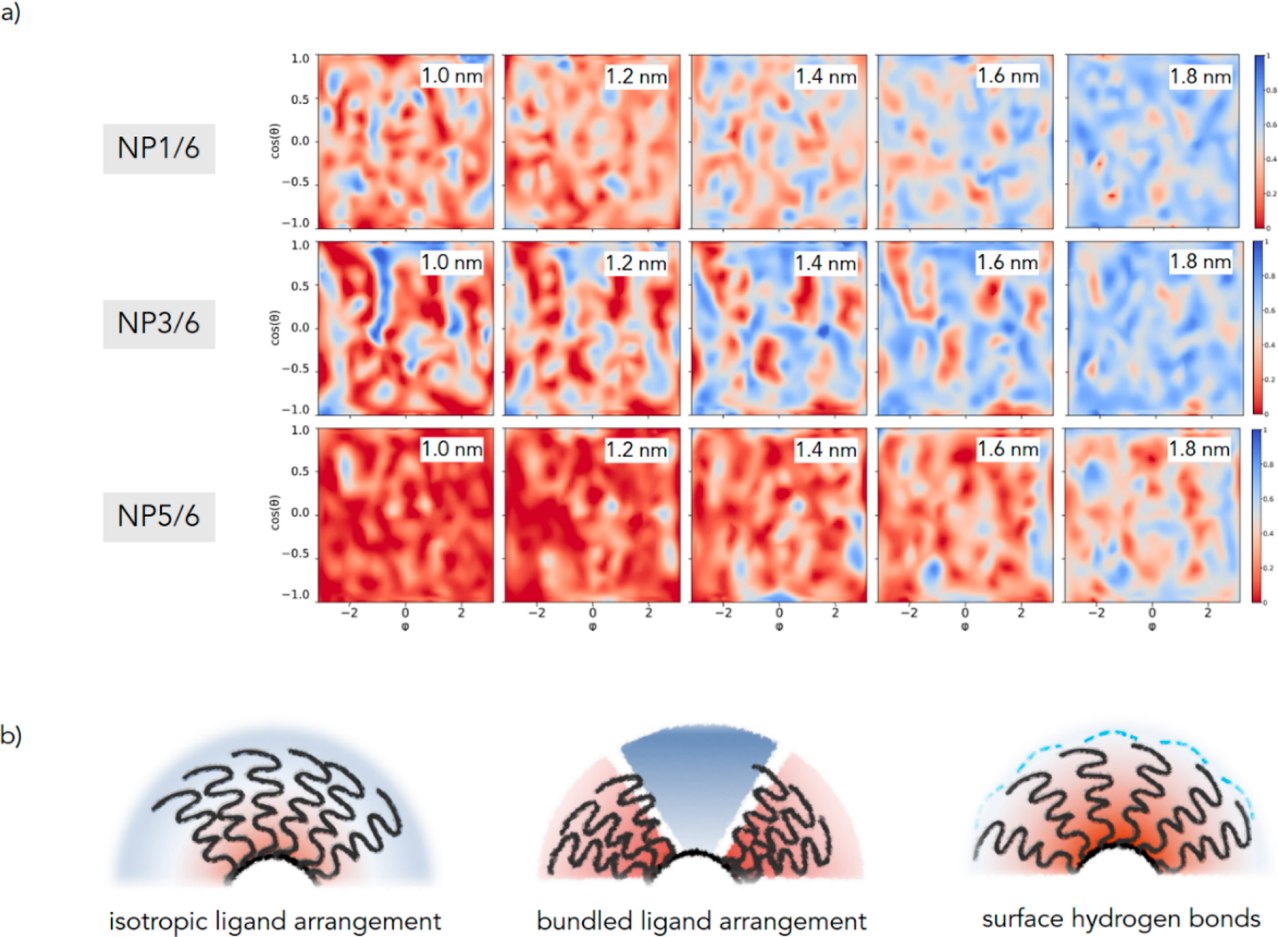
(a) Normalized water distribution at increasing distance from the gold surface for **NP1/6**, **NP3/6**, and **NP5/6**. The graphs plot the distribution of the atom (oxygen of water or carbon of thiolates) closest to gold surface (centered on the gold core and placed at increasing distances from its surface) shown as a two-dimensional projection of the sphere surface (*x*-axis, the azimuthal angle φ; *y*-axis, the cosine of the polar angle θ). A value of 1 indicates that an oxygen atom of a water molecule is always the closest; if it is equal to 0, it indicates that a carbon/fluorine atom of a chain is always the closest. Simplifying, red to salmon areas represent poorly hydrated zones, while blue areas stand for highly hydrated parts of the monolayer (at a certain distance from the gold surface). At distances lower than those considered, the microenvironment is almost hydrophobic, while at higher distances, it is fully hydrated and no major difference between the monolayers could then be detected. Maps for **NP2/6** and **NP4/6** can be found in the SI (Figures S5 and S6). (b) Examples of possible different hydration states within SAMs.

Revealing monolayer structure and molecular order is the first necessary step to gather information about nanoparticle hydration and solvation-related properties.^45^ Previous experimental and computational efforts^46−48^ have highlighted that ligand ordering is more correlated than other conventionally considered chemical properties (such as the solvent-accessible surface area (SASA)) with the interfacial hydrophobicity of SAMs. Most of the studies have been conducted on planar SAMs and, when extended to curved surfaces, were focused only on the description of the SAM–water interface. Here, we expand the investigation of nanoparticle hydration to the whole interior of the monolayer and we also consider the consequences of having mixtures of ligands bearing different hydrophobicities. To do that, we relate the normalized water content and the spatial distribution of water molecules within the monolayer at increasing distances from the core and we project it onto bidimensional planes. This provides an immediate view of the average degree of solvation of the monolayer and the topological distribution of the solvent within the monolayer (Figure 4).

When one observes the water density maps reported in Figure 4, it appears that isotropic monolayers allow a uniform diffusion of the solvent within the organic layer; the water content decreases progressively when moving toward the nanoparticle center, and there is a concentration gradient with respect to bulk solution (Figure 4b). On the contrary, the presence of bundles generates alternation of highly hydrated zones between the bundles (at a level comparable to that of bulk solvent) and dehydrated areas, where solvent penetration is hindered by the strong self-association among bundled alkyl chains.

An additional element affecting the hydration is the existence of the extended ligand/water hydrogen bond network on the nanoparticle surface, which reduces the internal diffusion of the solvent and makes zwitterionic nanoparticles less hydrated than other isotropic systems, like for example **NP1**.

Fluorine-rich ligands **6** are considerablely shorter than all the other thiolates; thus, when they segregate in domains, they enable the local diffusion of the solvent closer to gold, resulting in a higher content of water with respect to homoligand AuNPs at the same distance from the gold surface (see also Figures S5–S7).

### Automated Detection of Local (Supra)Molecular Environments in SAM

**Figure 5:**
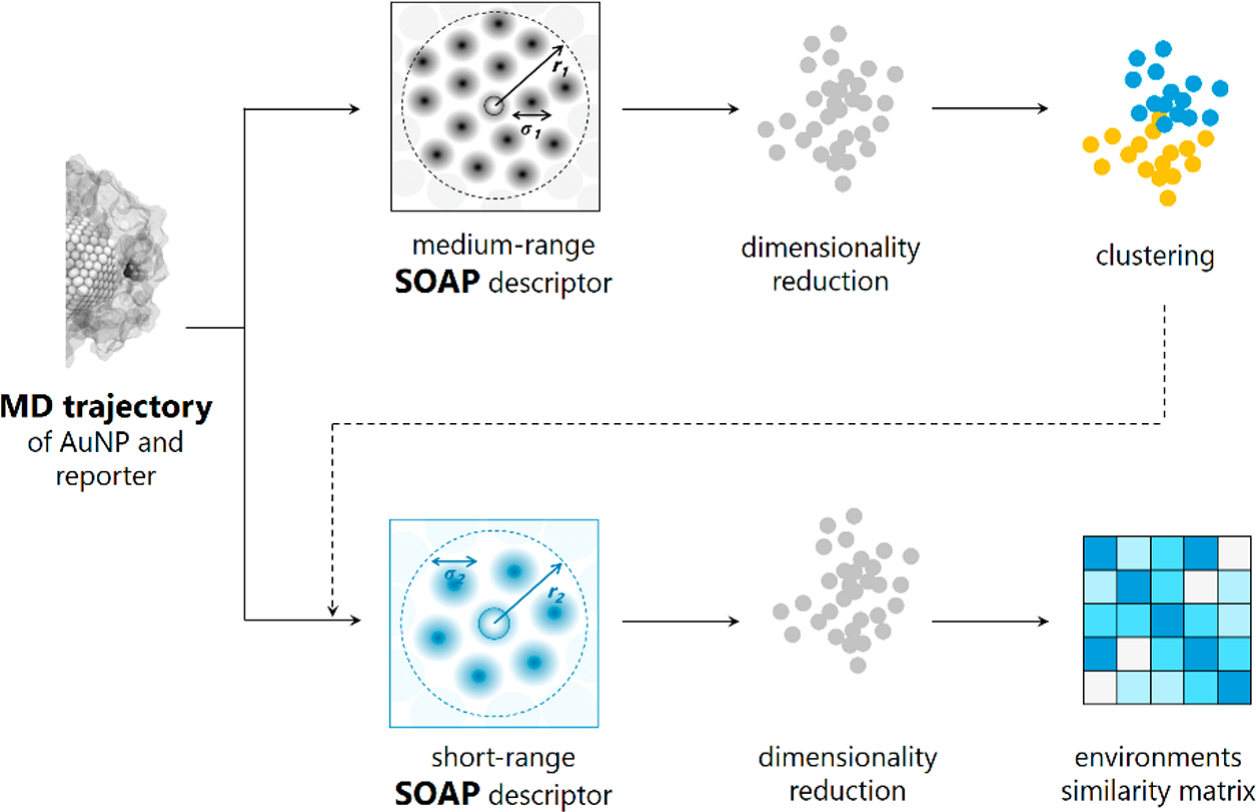
Conceptual diagram of the workflow used for the detection and comparison of local molecular environments within self-assembled monolayers (SAMs) using the Smooth Overlap of Atomic Positions (SOAP)-based structural analysis. Molecular dynamics calculations of the SAM-AuNP and reporter **7** are conducted in explicit solvent. The SOAP descriptor vector is constructed taking the reporter atoms (here the nitrogen atom) as the center of the structural environment up to a given cutoff radius *r*_1_ (*medium*-range description) and employed for the identification of molecular fingerprints assigned by an unsupervised clustering algorithm (step 1). The *short*-range SOAP descriptor is built considering only solvent molecules up to a range of *r*_2_ (<*r*_1_), and a linear kernel between SOAP vectors is used to measure the similarity between the environments (step 2) and interpreted by correlating the location of the data with the MD evidence. For more details on each step, see Figure S8 and Section S3 in the SI.

The calculations just described are the entry points of an automated workflow able to identify first and then compare local (supra)molecular environments within any SAMs. It is based on the combination of AA-MD calculations of SAM-AuNP carried out in explicit solvent, an agnostic machine-learning structural analysis employing the SOAP^49^ formalism to describe the 3D atomic environment that surrounds a reporter molecule (here, the radical probe **7**; see Figure 2) interacting with the monolayer, and an unsupervised probability-based method for clustering the data (Figure 5). In the SOAP framework, the local atomic environment of an atom (defined as a SOAP center) is represented by the sum of element-specific smooth Gaussian densities centered on the positions of neighborhood atoms within a spatial cutoff, and it is associated with a vector, commonly known as “SOAP power spectrum” or “SOAP fingerprint” (see SI Section S3 for the SOAP formal derivation). SOAP vectors provide a high-dimensional, agnostic representation of molecular environments. SOAP descriptors have been successfully applied in exploring the conformational landscape of single molecules,^50^ recognizing local structural motifs^51^ and describing formation/dynamics of soft supramolecular fibers,^52^ returning a rich structural/dynamical characterization of complex molecular systems. Such an analysis in our systems allows us to unveil different states of the molecular reporter **7** based on differences in the local environment (microenvironment) that surrounds it during the AA-MD simulation time, accounting for overall atomic composition, molecular conformation, local order, persistency in the interactions, and degree of solvation.

The workflow consists of two main steps both starting from an (equilibrated) AA-MD trajectory of a specific SAM-AuNP/**7** complex. The first one is the *classification* of the local states of the probe (step 1) (Figure 5 and flowchart in Figure S8). To identify the relevant microenvironments visited by **7**, the SOAP descriptors are calculated to be centered on the nitrogen atom of the probe. The SOAP data set includes all atoms within a cutoff radius *r*_1_ (9 Å), which is taken as a compromise between the ability to capture relevant local structural correlations and necessity to minimize the computational requirements for SOAP manipulation and storage (see SI Section S3, Figure S23). We refer to that as “medium-range SOAP vector”.

Linear principal component analysis (PCA) is then applied to reduce the high dimensionality of the SOAP features space (14354 dimensional on average) without losing important features. ∼94% of the total variance (e.g., global information) is retained keeping the first 10 principal components (Figure S24). Then, a probabilistic model based on Gaussian mixtures (GMMs) is exploited as an unsupervised clustering scheme. This allows one to partition and classify all the environments perceived by the probe into groups (i.e., clusters) and distinguish them without any prior information on the number of clusters (for a description of the clustering algorithm, see SI Section S3). The outcomes of PCA are visualized by projecting the 10 PCs in 2D onto the first two principal components, PCA1 and PCA2, to provide simple and intuitive maps.

**Figure 6:**
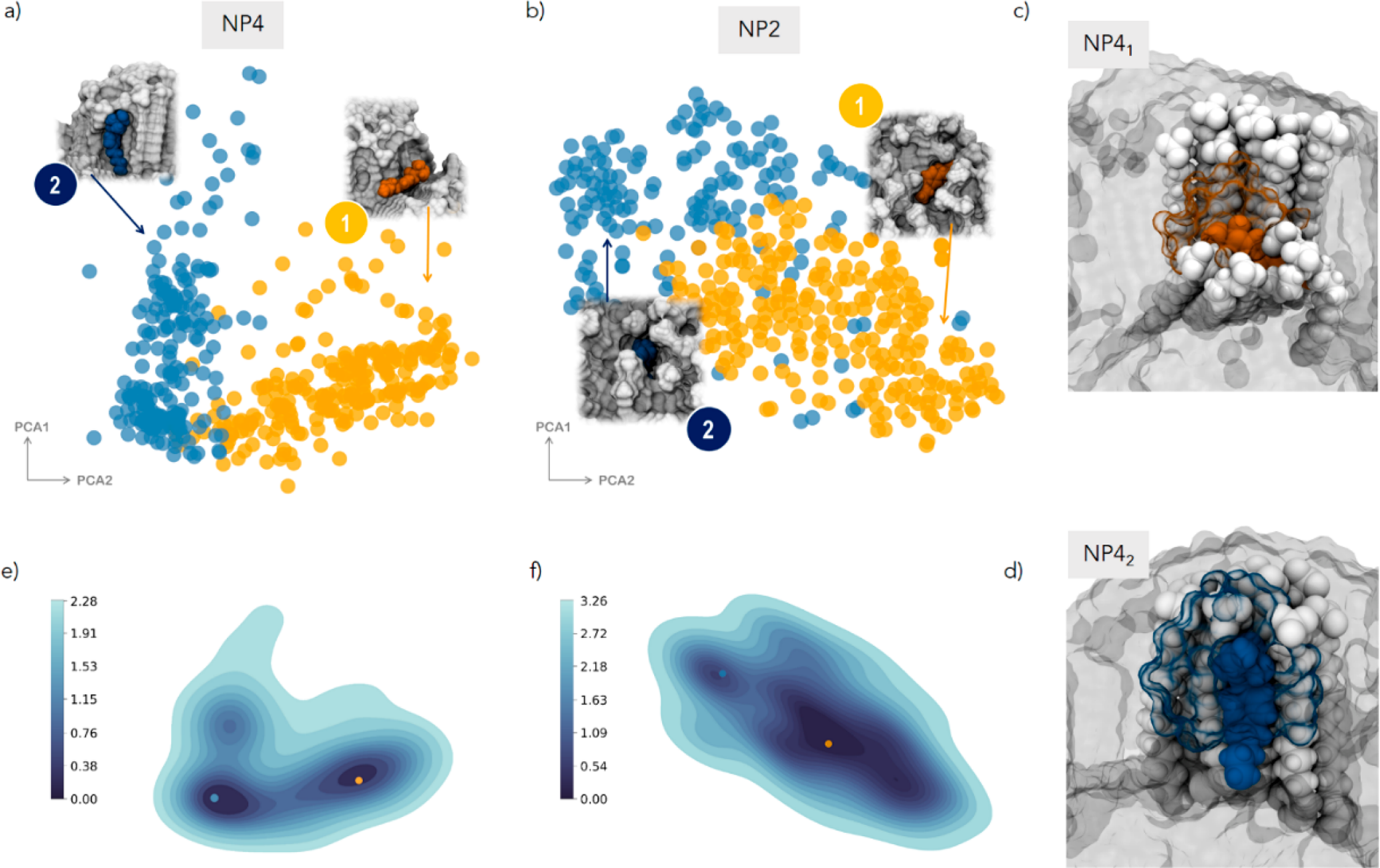
First two principal components (PCA1 and PCA2) obtained from dimensionality reduction of the medium-range SOAP feature space of the probe **7** in thicker homoligand **NP4** (a) and **NP2** (b). Dots are colored according to the clusterization obtained by the GMM analysis. For each cluster, the inset shows the molecular environment centered on the probe **7**, extracted from the corresponding MD frames. Color legend: probe, same color of the cluster; ligands **4** and **2** in gray; solvent not shown for clarity. (c, d) Example of the molecular view of the local environments **NP4**_**1**_ and **NP4**_**2**_ including all atoms within the cutoff *r*_1_. The reporter is colored according to the cluster assigned as a sphere; water is shown in the same color of the probe but as a transparent surface, and the ligands belonging to the environment are highlighted as white spheres. The remaining ligands are left as a background gray surface. (e, f) Free energy surface (FES) (kcal/mol) calculated from the state’s probability distribution in (a) and (b), respectively. Dots identify the minima on the FES and are colored based on the microstate (cluster) they refer to.

Through the SOAP-GMM analysis, two distinct states (i.e., microenvironments) are identified for **NP4** (Figure 6a): the probe **7** lays at the ligand bundle–water interface close to the gold core (1, orange) or parallel to ligand chain (2, blue). From now on, each local environment is reported in subscript: for example, **NP4**_**1**_ indicates the local environment (1) in **NP4**.

As an example, Figure 6c,d shows a molecular view of the ligands and water molecules forming the local environments **NP4**_**1**_ and **NP4**_**2**_. From the MD trajectory, we also calculate the free energy surface (FES) of the reporter **7** in the system as the probability distribution of states in the PCA reduced SOAP feature space (P) by using the standard statistical relation FES = −*K*_b_*T* log(*P*) and find that the states correspond to two local minima equally visited by **7** (Figure 6e). The classification is fully consistent with our previous findings,^28^ where two distinct positions of **7** were also identified by classical analysis of the MD trajectory in **NP4**, one more deeper in the organic layer and one more exposed to the exterior.

Two structural states are also detected in **NP2** (Figure 6b), meaning that thicker monolayers are able to host a small molecule in structurally distinguishable *loci*. Yet, inspection of the clusterization maps suggests that the difference between the two states is sharper in bundled shells. In fact, in **NP4**, the clusters are well distinct and clearly separated; in **NP2**, the transition is smoother, although measurable by SOAP-GMM. We attribute this to the diverse ligand arrangement in **NP2** and **NP4**. Chain packing allows accommodation of a small molecule like **7** by simple binding at the ligand bundle interface at increasing depth from the outer surface, and the search of an optimal interaction position for the probe is facilitated by the freedom to explore the conformational space at that interface; this would lead to well-defined binding sites for **NP4**.

On the other hand, in isotropic monolayers as **NP2**, the accommodation of a guest requires diffusion within the ligands, hampering the access to the whole depth of the monolayer and thus leveling out differences between interaction positions.

When the SOAP-GMM classification is applied to nanoparticles having a shorter hydrophobic portion like **NP1** and **NP3**, one single microenvironment is identified for **7** (see Figure S9). Although **NP5** could be assimilated to **NP1** and **NP3**, the classification returns a different picture (Figure S9); in fact, it unveils the presence of two clusters, namely, two states explored by the probe. Nevertheless, the FES indicates that one of them is much more visited than the other and sets itself as a local minimum. The more complex behavior of the zwitterionic **NP5** reflects the uniqueness of this monolayer in agreement with the evidence from the AA-MD calculations.

**Figure 7:**
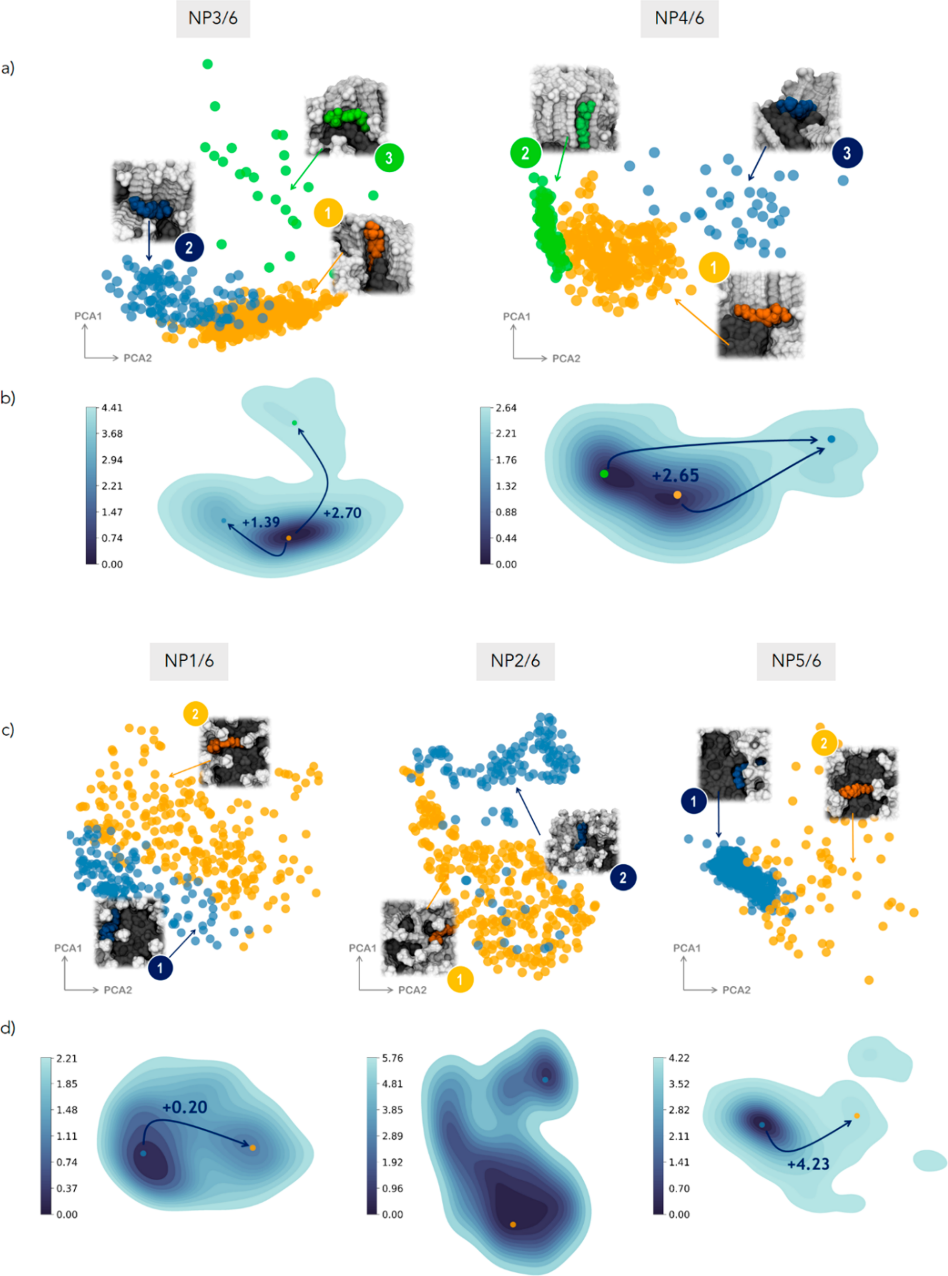
First two principal components (PCA1 and PCA2) obtained from dimensionality reduction of the medium-range SOAP feature space of the probe **7** in heteroligand bundled **NP3/6** and **NP4/6** (a) and isotropic **NP1/6**, **NP2/6**, and **NP5/6** (c) monolayers. Dots are colored according to the clusterization obtained by the GMM analysis. For each cluster, the inset shows the molecular environment centered on the probe **7**, as extracted from the corresponding MD frames. Color legend: probe, same color of the cluster; ligands **1**–**5** colored in gray; ligand **6** colored in dark gray; solvent not shown for clarity. (b) Free energy surface (FES) (kcal/mol) calculated from the state’s probability distribution for **NP3/6** and **NP4/6** (b) and **NP1/6**, **NP2/6**, and **NP5/6** (d). Dots identified the minima on the FES and are colored based on the microstate (cluster) they refer to. The arrows indicate the transition probabilities between the states from the minimum.

Mixed shells containing hydrophobic patches enrich the probe state space compared to their respective homoligand nanoparticles. There is a marked difference between bundled and isotropic monolayers (Figure 7).

**NP3/6** and **NP4/6**, which have ligand clusters, show three (metastable) local environments for the probe: the first on the fluorinated chains (**NP3/6**_**3**_ and **NP4/6**_**3**_) being the least visited, the second down at the interface between alkyl and fluorinated domains (**NP3/6**_**2**_ and **NP4/6**_**1**_), and the third with the probe parallel to the bundles (**NP3/6**_**1**_ and **NP4/6**_**2**_). The three states are distinct and clearly separated in the SOAP feature space. On the contrary, in isotropic monolayers like **NP1/6** and **NP2/6**, there are only two states possible, which are not so well divided in the SOAP space as in **NP3/6** and **NP4/6**, thus highlighting the importance of the monolayer arrangement in shaping local environments. An exception is the zwitterionic **NP5/6** for which the interfacial **NP5/6**_**1**_ is highly favorable and well distinguished from **NP5/6**_**2**_. From the FES inspection, still in heteroligand monolayers, two states are the most probable for C_16_ long chains (i.e., **NP2/6** and **NP4/6**), and these reduce to one for shorter ligands (**NP1/6**, **NP3/6**, and **NP5/6**).

### Comparison of Local Environments in Different SAMs

Once the most probable interaction site(s) is identified for each system, we want to *compare* them (step 2 of the workflow, Figure 5). This means assessing how much those local environments are similar; they belong to either the same monolayer or to different nanoparticles. To ensure that the SOAP analysis is meaningful and fully comparable across different systems, one needs to choose a representation of the structural space that takes into account common features between systems. For that reason, we select the (roughly) first hydration layer of the reporter **7**. This choice also allows us to limit the computational costs, since now all the systems have to be analyzed together. The nitrogen atom of the probe **7** is still assigned as the SOAP center, and the cutoff radius *r*_2_ is now set to 4.5 Å (Figure S23), including only solvent molecules. We refer to that as “short-range SOAP”. Accordingly, taking the MD snapshots where the probe is in the most favorable state(s) based on the assignment of the medium-range SOAP-GMM clusterization, we construct the corresponding short-range SOAP fingerprint for each molecular environment and each nanoparticle. Then, we perform a dimensionality reduction via linear PCA to obtain a low-dimensional representation and consider only the first 10 components (Figure S24).

**Figure 8:**
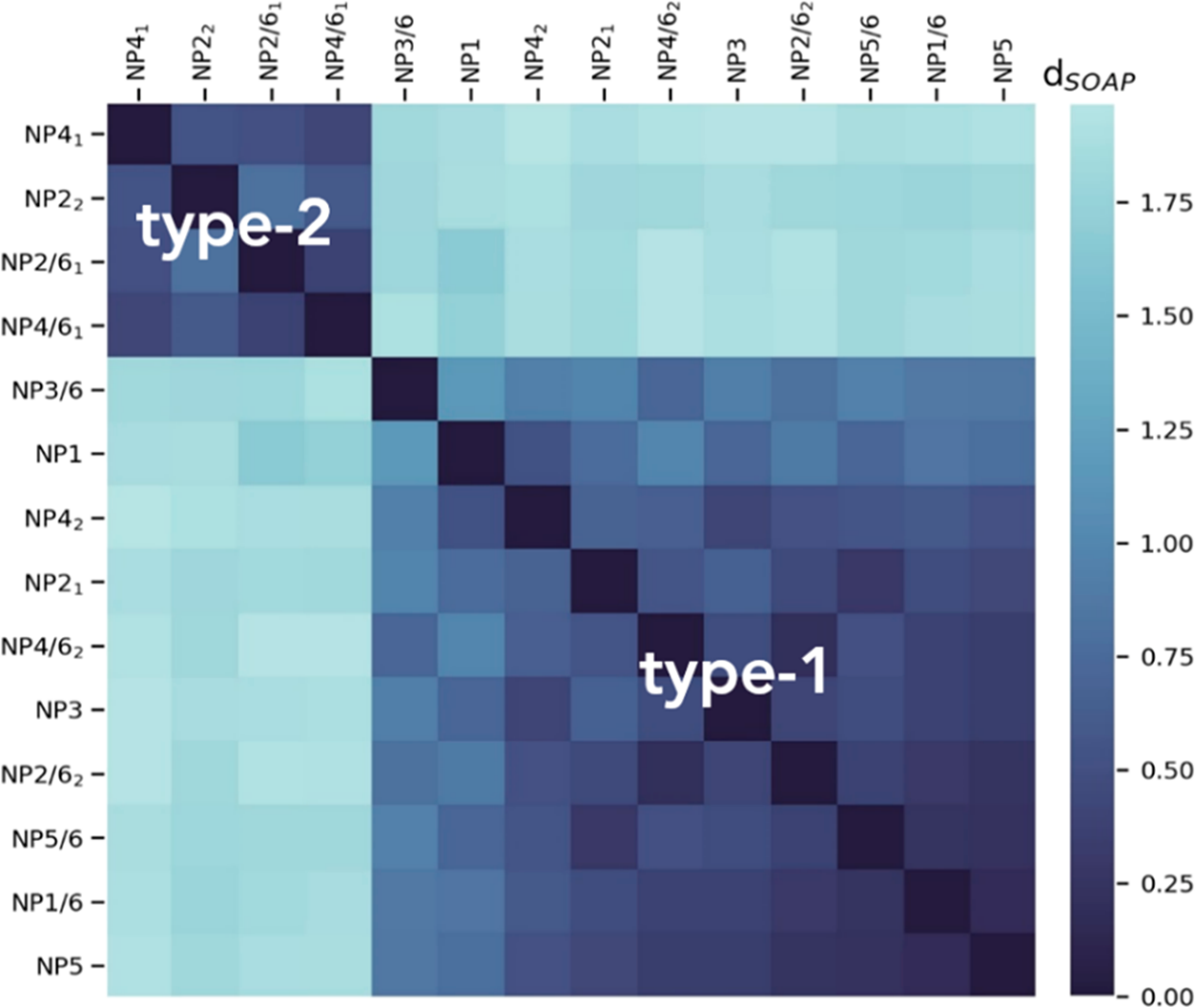
Similarity matrix for all local (most visited) environments generated by calculating the pairwise SOAP kernels *K*_SOAP_ between all the reduced short-range SOAP feature vectors. Dark blue color indicates high similarity between the environments.

Measuring structural similarity requires the definition of a metric that is capable of identifying identical molecular fingerprints. There are different ways of combining atom-centered representations to obtain a structure-level comparison;^53^ in the SOAP space, one natural choice is to define a linear kernel of the density representation in the form of the dot product of the SOAP power spectra of the two molecular environments *K*(*i*,*j*)_SOAP_ (see SI Section S3). SOAP-based structural similarity kernels can be interpreted as a measure of how much two (smoothed) atomic distributions (*i*,*j*) are superimposed on each other (i.e., how much similar the local environments are in the SOAP space). The data are displayed in the form of a similarity matrix by converting the similarity value *K*(*i*,*j*)_SOAP_ to an Euclidean distance metric *d*_SOAP_, ranging from 0 to 2 (Figure 8).

The color level is proportional to the value of the similarity between environments: dark blue corresponds to the highest value of similarity computed and light blue indicates larger SOAP distances and an increased structural difference between the environments. From Figure 8, it appears evident that there are two distinct classes of environments according to the similarity metric: the first one (type-1) is that corresponding to the lower right quadrant of the matrix and the second (type-2), to the upper left quadrant.

It is worth noting that the classes are not equally populated. Type-2 includes only a few molecular environments, of which all exist in thick monolayers (namely, **NP2**- and **NP4**-type nanoparticles), while the type-1 class is broader and comprises environments from long (−S–C_16_–FG) to short (−S–C_12_–FG) chain shells. This interesting piece of evidence suggests that thick (≥C_16_) monolayers have the ability to form local environments with structural features well distinguishable from those existing in thin monolayers. In addition, the results show that it is possible to capture and discriminate multiple environments applying a pure data-driven evaluation without *a priori* assumptions.

To gain more insights and in an attempt to rationalize these outcomes, we then link each state to the corresponding molecular structure retrieved from the MD snapshots as assigned by the medium-range SOAP-GMM to that environment; in this way, we find out that nanoparticles with a single interaction site (namely, −S–C_12_–FG) are classified as type-1; in systems with two main interaction sites, one is of type-1 and the other is of type-2. Type-2 sites correspond to local environments where the probe is placed closer to the gold core and the overall hydration is limited (as an example, see Figure 6c for **NP4**_**1**_ or Figure 7a for **NP4/6**_**1**_). We assess that by simply calculating the radial distribution function (RDF) of the nitrogen atom of the reporter (i.e., the probability distribution as a function of distance from the metal center) from the corresponding MD frames and matching the peak of the RDF with the solvation map to that distance. Type-1 environments instead share a higher solvation, and the probe is more exposed to the external environment (as an example, see Figure 6d for **NP4**_**2**_ or Figure 7a for **NP4/6**_**2**_). The chemistry of the thiolates end group has no major influence on the features of the interaction site, which is not completely surprising since the probe is mainly interacting with the alkyl part of the ligands (Figures 6, 7, and S9).

### ESR Analysis of SAM-AuNPs

Experimentally, monolayer features can be investigated by molecular probes, which are able both to enter inside the monolayer and to possess spectral features that depend on the molecular environment of the surroundings. Functionalized benzyl *tert*-bytulnitroxides (BTBN) possess such characteristics and have been largely employed to characterized different types of water-soluble SAM-protected AuNPs.^54−57^ In the present study, probe **7** containing a pentyl chain at the *para* position of the aromatic ring and a hydroxymethyl group in place of the methyl in the *tert*-butyl substituent is employed for ESR investigation. This hydrophobic probe has been chosen because of its good affinity for the nanoparticle organic monolayer when dissolved in water. Experimental values of hyperfine splitting constants (hfsc’s) of heteroligand nanoparticles are collected in Table 1 together with those previously^28^ measured in the presence of homoligand nanoparticles and in a temperature range between 300 and 340 K (for details on mixed-monolayer nanoparticle synthesis, XPS characterization, and ESR measurements, see the Experimental Section and SI Sections S2, S4, and S5; otherwise, refer to our previous work^28^).

**Table 1:**
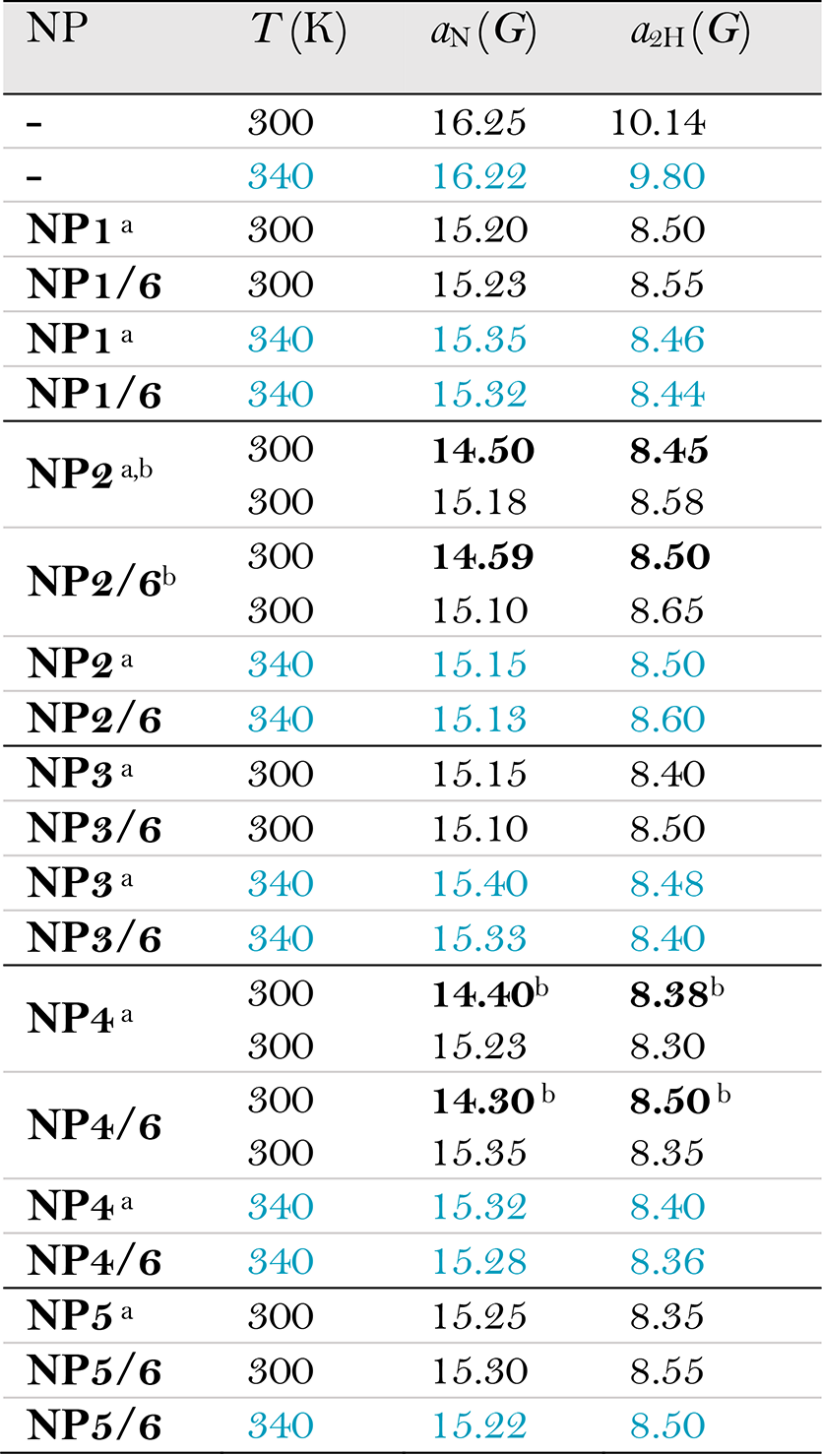
Spectroscopic Parameters for Radical Probe **7** at Different Temperatures (Black at 300 K and Light Blue at 340 K)

a Data from ref (28).

b The *a*_N_ values given in bold refer to the probe in the most hydrophobic location.

**Figure 9:**
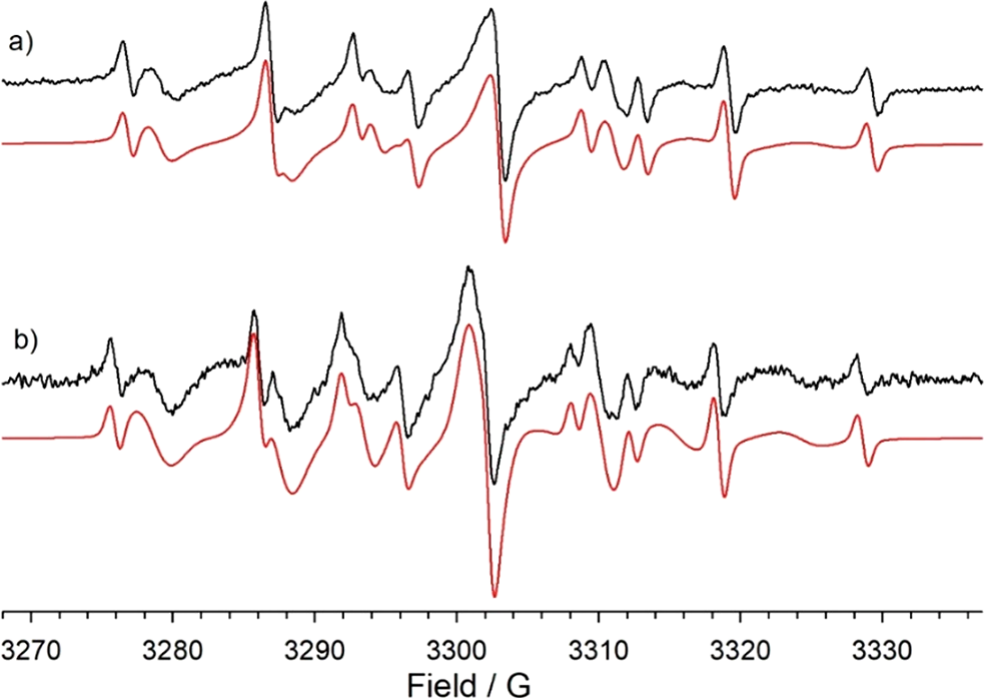
ESR spectra of the radical probe **7** recorded in water in the presence of **NP3/6** (a) and **NP4/6** (b) at 300 K. In red are reported the corresponding theoretical simulations obtained by employing the spectroscopic parameters reported in Table 1.

With **NP1/6**, **NP3/6**, and **NP5/6** and their homoligand partner, spectra are characterized by two different resolved sets of signals (as an example, see Figure 9) at 300 K. The one with larger hyperfine coupling constants is due to the probe located in water, while the second one, has nitrogen hfsc (*a*_N_, reported in Table 1) significantly smaller than that measured for **7** in solution, resulting from the probe positioned in the monolayer. Analysis of the spectra suggests the presence of a single interaction site, in line with the SOAP-GMM analysis for nanoparticles having short chain shells.

In all thick monolayers (namely, **NP2**- and **NP4**-type systems), the ESR analysis shows the presence of two distinct environments, where the probe bound to the monolayer experiences different background polarities (Table 1). The first one has experimental values of *a*_N_ in the range of 14.30–14.60 G, significantly smaller than that for the probe in water (16.26 G), indicating an extremely low polarity. This is consistent with type-2 settings, where **7** lies close to the gold core and the overall hydration is limited, thus corresponding to **NP2**_**2**_, **NP4**_**1**_, **NP2/6**_**1**_, and **NP4/6**_**1**_ states. In addition, such low values of *a*_N_ are seen only for long chain shells, in agreement with the SOAP-GMM classification (see Figure 8).

The second interaction location has much higher spectroscopic parameters (15.10–15.35 G), closer to that of the probe free in solution, which are indeed associated with an increased environment polarity perceived by the probe. The existence of a second interaction site in **NP2**- and **NP4**-type systems parallels well the computational prediction, supporting the possibility to have distinguishable local environments within the same (thick) monolayer. Interestingly, the spectroscopic parameters are comparable to those for **NP1**-, **NP3**-, and **NP5**-type monolayers. This shows that the radical samples very similar environments in all these systems. SOAP similarity analysis returns a classification which is in line with this interpretation: in fact, **NP2**_**1**_**NP4**_**2**_, **NP2/6**_**2**_, and **NP4/6**_**2**_ environments are assigned to the same category (type-1 class) as **NP1**(**/6**), **NP3**(**/6**), and **NP5**(**/6**) environments (Figure 8).

By increasing the temperature, a new set of signals, characterized by spectroscopic parameters very similar to those previously measured in type-1 *loci* appears in the spectrum, as was also seen by repeating the SOAP-GMM analysis including the most stable states at 340 K (Figures S10 and S11). Hence, when the temperature is increased, the probe experiences local environments with higher polarity, solvation, and exposure to the surroundings that makes type-1 sites the most favorable interaction locations for the systems under investigation.

Quite unexpectedly, based on our current understanding,^26,57^ the spectroscopic parameters of the probe in mixed-monolayer NPs do not differ significantly from those measured in the corresponding homoligand shell. Previous evidence on the monolayers made by mixtures of hydrocarbon/perfluorocarbon chains terminating with a short poly(oxoethylene) moiety indeed suggested that the probe should preferentially reside in fluorinated domains. Here, instead, MD calculations clearly show that **7** never enters or fully interacts with the fluorinated patches and is located preferably at the fluorine domain interface (and thus explains the similarity in the nitrogen hspcs). In addition, MD calculations and Voronoi diagrams display that short F-alkyl chains are densely packed in the NPs considered here, physically and energetically preventing them to host the radical probe.

## Conclusions

In summary, ligands self-assembling on the surface of gold nanoparticles can create local (supra)molecular environments with unique fingerprints that allow them to be precisely detected and exploited. We have presented a computational approach, which enables automated identification and comparison of such environments driven from the data (i.e., from atomistic MD trajectories) and without feeding input parameters. The computational workflow is built on unsupervised clustering of the SOAP atomic descriptors and a simple SOAP metric to classify the environments. In this proof-of-concept study, we have considered a collection of chemically different SAM-AuNPs, bearing cationic, anionic, and zwitterionic surface groups and having different monolayer thicknesses. The set includes homo- and heteroligand monolayers; the second ones present alternating hydrophilic/hydrophobic surface patterns that stem from the nanoscale separation of two immiscible ligands. By the SOAP analysis and in conjunction with ESR measures, we have successfully demonstrated that multiple structural and chemical microenvironments can exist together within the SAM-AuNPs investigated. In particular, they differ for accessibility, local solvation, and hydrophobicity, which are imparted by specific ligand length, nature of the ligand end group, and monolayer 3D structure.

The results of our investigation allow us to draw some general conclusions: (i) anisotropic monolayers may facilitate the establishment of settings having well-defined and easily distinguishable local (supra)molecular motifs; (ii) in the absence of chemical groups designed to recreate specifically intended binding or catalytic sites, thick monolayers naturally lead to multiple, coexisting environments, which are shaped by confined solvent, organization, and conformational mobility of the ligands; (iii) surface patterns in heteroligand shells give rise to a multiplicity of states, which could be potentially targeted under appropriate thermodynamic or kinetic pathways.

Overall, this work provides a promising general approach for systematic and computationally efficient investigation of local (supra)molecular environments in SAM-AuNPs, a widely used class of O–I nanomaterials, and establishes a mechanistic understanding of their intimate features with a full account of nanoscale effects. The next steps will be the extension to more complex functional nanoparticles and the design guided by machine-learning algorithms of local motifs with predefined properties.

## Experimental Section

### Nanoparticle Synthesis and Characterization

Detailed synthetic procedures and characterization for mixed monolayers nanoparticles can be found in the Supporting Information; otherwise, the reader may refer to our previous work.^28^ All commercial reagents were purchased from Aldrich and VWR and used without purification unless otherwise mentioned. Solvents were purchased from Aldrich and VWR and deuterated solvents, from Cambridge Isotope Laboratories and Aldrich. Dry solvents were obtained from Aldrich. Chlorinated solvents were kept over K_2_CO_3_ for at least 24 h prior to use. All other solvents were reagent grade and used as received. Reactions were monitored by TLC on Merck silica gel plates (0.25 mm) and visualized by UV light, I_2_, or KMnO_4_–H_2_SO_4_ solution. Chromatography was performed on Merck silica gel 60F-254 (230–400 mesh), and the solvents employed were of analytical grade. NMR spectra were recorded on a Varian 500 spectrometer (operating at 500 MHz for proton and at 125 MHz for ^13^C) or on a Varian 400 MHz (operating at 400 for proton, at 376.16 MHz for ^19^F, and at 100.5 MHz for carbon). ^1^H NMR chemical shifts were referenced to the residual protons in the deuterated solvent. ^19^F NMR spectra were referenced to CFCl_3_ chemical shift, and ^13^C NMR chemical shifts were referenced to the solvent chemical shift. Chemical shifts (δ) are reported in ppm, and the multiplicity of each signal is designated by the conventional abbreviations: s, singlet; d, doublet; t, triplet; q, quartet; m, multiplet; br, broad; dd, doublet of doublets. Coupling constants (*J*) are quoted in Hz. UV–visible spectra were recorded on a Shimadzu UV-1800 spectrophotometer. TGA analyses were performed on TGA Q500 V6.3 Build 189 using a heating rate of 10 °C min^–1^ up to 1000 °C under N_2_ flow. TEM images were obtained with a Philips EM208 electron microscope operating at 100 keV using a Emsis Quemsa CCD camera. TEM samples of protected gold nanoparticles were prepared by placing a single drop of 0.5 mg mL^–1^ MeOH or H_2_O/iPrOH solution onto a 200-mesh copper grid coated with an amorphous carbon film. NP gold core diameters were measured manually using a Gatan software Digital Micrograph on at least 200 particles. Electrospray ionization (ESI) mass analyses were performed on a PerkinElmer APII at 5600 eV and exact mass analyses, on a Bruker Daltonics microTOF-Q operating at 3200 V capillary potential. DLS measurements have been performed on a Malvern zeta Sizer Nano using a concentration for the nanoparticles between 0.1 and 0.4 mg/mL in water, scattering angle of 173°, 25 °C, and disposable cuvettes.

### Molecular Modeling Methods

A coarse-grained (CG) simulation approach based on dissipative particle dynamics (DPD) was first adopted to retrieve the phase separation of ligands on a gold surface in mixed SAMs, namely, nanoparticles **NP1–5/6**. This choice was necessary since the self-organization of chains requires long times that cannot be accessed simply by atomistic calculations. Once obtained, the CG nanoparticle model was mapped back onto the corresponding all-atom (AA) nanoparticle structure. Homoligand SAMs were modeled purely at atomic level. The full computational procedure for constructing the CG and AA SAM-functionalized NPs follows our previous works^21,22,27,29,38^ and is described in detail in the Supporting Information. AA nanoparticle models in explicit water were then extracted from equilibrated MD trajectories and used for subsequent MD and SOAP-GMM analysis. CG calculations were carried out in a Culgi simulation package (v.12.0, Culgi B.V., Leiden, The Netherlands) and AA simulations, in an AMBER 18 modeling suite.

### MD and SOAP-GMM Analysis

MD analysis was generated with a combination of an AMBER analysis tool, in-house developed Python codes, and Python package *scipy*.^58^ SOAP descriptors were derived by using the *Dscribe*(59) Python package. For GMM clusterization and environment classification, we adopted the *scikit-learn*(60) Python package. The parameter setting is given in the Supporting Information.

### Electron Spin Resonance (ESR) Measurements

ESR spectra were collected using a Bruker ELEXYS spectrometer equipped with an NMR gaussmeter for field calibration. The sample temperature was controlled with a standard variable temperature accessory and monitored before and after each run using a copper-constantan thermocouple. The instrument settings were as follows: microwave power 5.0 mW, modulation amplitude 0.05 mT, modulation frequency 100 kHz, and scan time 180 s. Digitized ESR spectra were transferred to a personal computer for analysis using digital simulations carried out with a program developed in our laboratory and based on a Monte Carlo procedure.

### Synchrotron-Based X-ray Photoelectron Spectroscopy (XPS) Measurements

Synchrotron-based X-ray photoelectron spectroscopy (XPS) experiments were carried out at the Material Science beamline of the Elettra synchrotron radiation facility in Trieste, Italy. The NPs were dispersed in aqueous solution and then drop-casted on a *n*-doped Si wafer, capped with a layer of native oxide (thickness of the oxide ∼4 nm). After drying the samples for 24 h in a protected environment at atmospheric pressure, they were inserted in the experimental UHV chamber of the beamline and promptly measured. The base pressure during the experiment was ca. 2 × 10^–10^ mbar. XPS spectra were acquired by means of a Specs Phoibos 150 mm mean-radius electron energy analyzer, equipped with a 1D-delay line detector built in-house. The overall energy resolution of the experiment was ca. 200 meV. The photoelectrons were collected at a normal emission angle, and for each sample measured, the same acquisition conditions (pass energy, entrance slit, lens mode of the spectrometer) were used. The measured signal was normalized to the photon flux and to the number of sweeps. The decomposition of the core-level spectra was carried out by using Doniach–Sunjic profiles^61^ convoluted with a Gaussian (to take into account the experimental resolution and the effects of thermal and inhomogeneous broadening) on a linear background, thus obtaining the line shape parameters, the photoemission intensity (i.e., the area delimited by the peak), and the core electron binding energy (BE) for each spectral component.

## Supporting Information Available

Additional computational and experimental results, synthesis and characterization of mixed-monolayers nanoparticles, computational methods, ESR background, and details on XPS analysis (PDF)
