# Supplementary material for: Spotting Local Environments in Self-Assembled Monolayer-Protected Gold Nanoparticles
Source: ACS Nano. 2022 Dec 2;16(12):20902–14. doi: 10.1021/acsnano.2c08467 (PMC9798909; doi:10.1021/acsnano.2c08467)
Supplement: Supplementary file 1 — nn2c08467_si_001.pdf [file nn2c08467_si_001.pdf]

SUPPORTING INFORMATION

Spotting Local Environments in Self-Assembled Monolayer-Protected Gold Nanoparticles

Cristian Gabellini,[a] Maria Sologan,[b] Elena Pellizzoni,[b] Domenico Marson,[a] Mario Daka,[b] Paola Franchi,[c] Luca Bignardi,[d] Stefano Franchi,[e] Zbyšek Posel,[f] Alessandro Baraldi,[d] Paolo Pengo,[b] Marco Lucarini,[c,*] Lucia Pasquato[b,*] and Paola Posocco[a,*]

[a]Department of Engineering and Architecture, University of Trieste, 34127 Trieste (Italy)
[b]Department of Chemical and Pharmaceutical Sciences and INSTM Trieste Research Unit, University of Trieste, 34127 Trieste (Italy)
[c]Department of Chemistry "G. Ciamician", University of Bologna, I-40126 Bologna (Italy)
[d]Department of Physics, University of Trieste, 34127 Trieste (Italy)
[e]Elettra Sincrotrone Trieste, 34149 Basovizza, Trieste (Italy)
[f]Department of Informatics, Jan Evangelista Purkyně University, 400 96 Ústí nad Labem (Czech Republic)

*corresponding author: paola.posocco@dia.units.it; l.pasquato@units.it; marco.lucarini@unibo.it

TABLE OF CONTENTS

S1. ADDITIONAL COMPUTATIONAL AND EXPERIMENTAL RESULTS.. 3

S2. SYNTHESIS AND CHARACTERIZATION OF MIXED-MONOLAYERS NANOPARTICLES 12

S2.1. Synthesis of NP-C12N/F6 (NP1/6) 12

S2.2. Synthesis of NP-C16N/F6 (NP2/6) 13

S2.3. Synthesis of NP-ZWPN/F6 (NP5/6) 15

S2.4. Synthesis NP-MDDS/F6 (NP3/6) 17

S2.5. Synthesis NP-MHDS/F6 (NP4/6) 19

S3. COMPUTATIONAL METHODS 22

S3.1. Dissipative particle dynamics (DPD) 22

S3.2. Molecular dynamics (MD) 24

S3.3. Voronoi tessellation and area dispersion index (ADI) 26

S3.4. Smooth Overlap of Atomic Position (SOAP) 26

S3.5. Gaussian mixtures clustering algorithm 28

S3.6. Similarity measure 30

S4. ELECTRON SPIN RESONANCE (ESR) 32

S5. SYNCHROTRON-BASED X-RAY PHOTOELECTRON SPECTROSCOPY (XPS) 33

S6. REFERENCES 34

S1. ADDITIONAL COMPUTATIONAL AND EXPERIMENTAL RESULTS

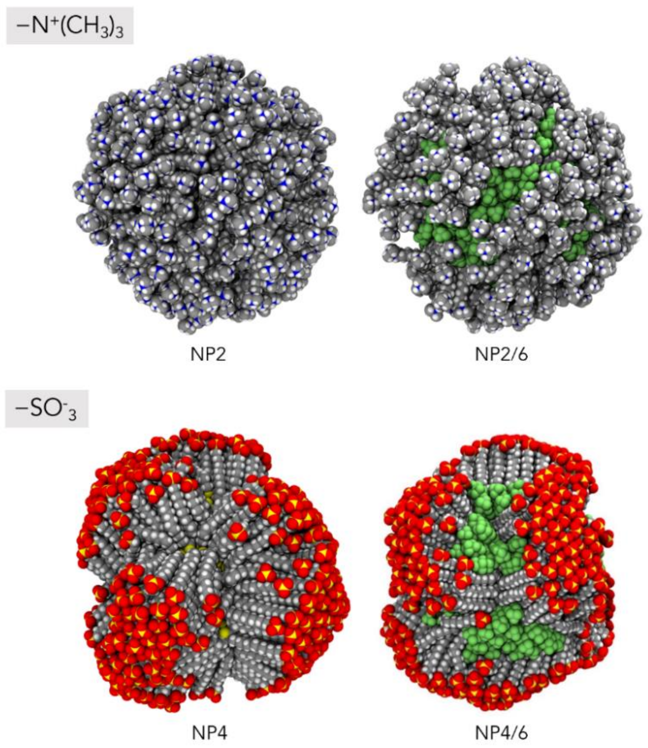

Figure S1. Representative molecular structures of homoligand **NP2**, **NP4** nanoparticle and its heteroligand **NP2/6**, **NP4/6** counterpart from molecular dynamics simulations in explicit solvent (water). For clarity, water and counterions are not shown. Color representation of atoms: C, grey; O, red; S, yellow; N, blue; F, green; H, white.

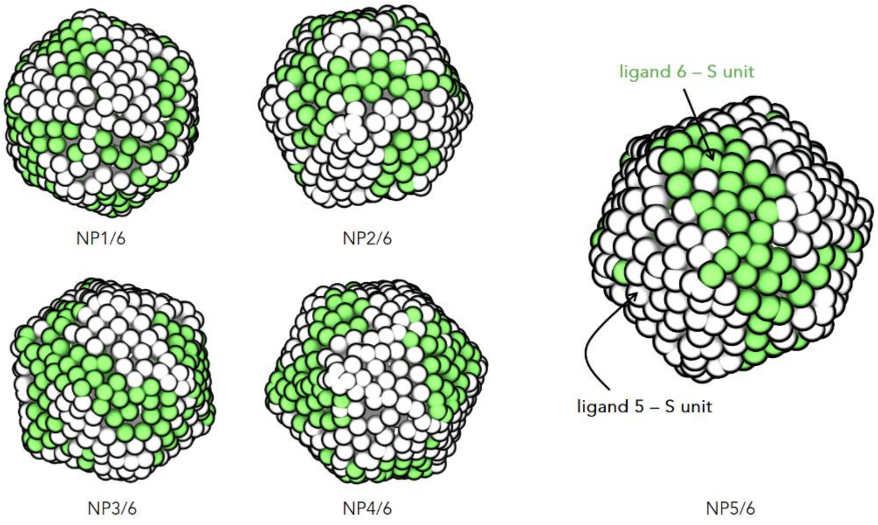

Figure S2. Mesoscale equilibrium arrangement of sulfur (S) units in heteroligand nanoparticles from DPD simulations. Green spheres represent sulfur beads belonging to ligands **6** and white spheres to ligands **1**, **2**, **3**, **4**, **5**, depending on the system under consideration.

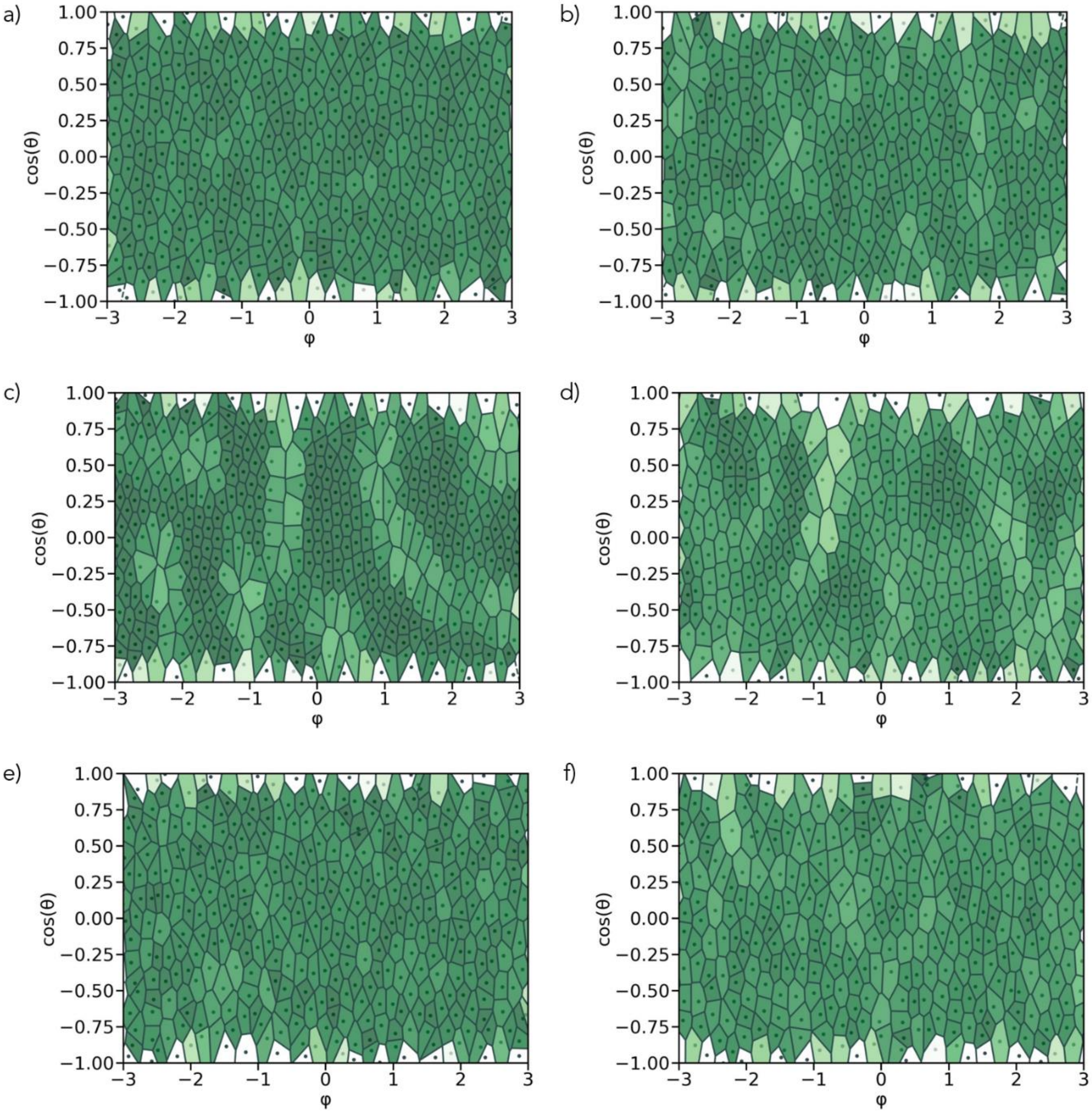

Figure S3. Voronoi tessellation based on the center of mass of the ligands projected onto a bi-dimensional (φ, cos (θ)) plane for the shorter alkyl chain (C_{11}/C_{12}) nanoparticles. Each dot corresponds to a ligand center of mass. Each polygon is colored according to its value, where darker (lighter) regions represent smaller (larger) area with higher (lower) ligand density with respect to the average. a) **NP1**, b) **NP1/6**, c) **NP3**, d) **NP3/6**, e) **NP5**, f) **NP5/6**.

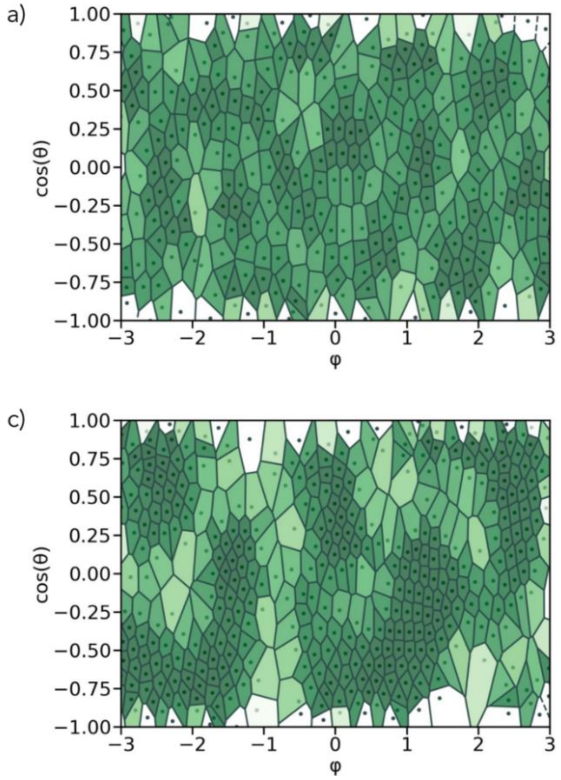

Figure S4. Voronoi tessellation based on the center of mass of the ligands projected onto a bi-dimensional (φ, cos (θ)) plane for the longer alkyl chain (C_{16}) nanoparticles. Each dot corresponds to a ligand center of mass. Each polygon is colored according to its value, where darker (lighter) regions represent smaller (larger) area with higher (lower) ligand density with respect to the average. a) **NP2**, b) **NP2/6**, c) **NP4**, d) **NP4/6**.

Table S1. Nanoparticle structural properties including: radius of gyration (Rg), average number of ligand bundles (n_b), asphericity (δ), fraction of *trans* dihedrals angles, area dispersion index (ADI) calculated from MD simulations. Uncertainties are reported in brackets.

	R_g (nm)	n_b[a]	δ[b] (-)	Fraction of *trans* dihedrals (%)[c]	ADI[d]
**NP1**	3.16 (0.02)	-	0.08 (0.03)	90.6 (3.0)	2.24
**NP1/6**	2.99 (0.04)	-	0.13 (0.04)	85.6 (3.3)	2.90
**NP2**	3.15 (0.05)	-	0.13 (0.04)	88.5 (6.1)	2.52
**NP2/6**	3.03 (0.05)	-	0.33 (0.07)	85.2 (5.6)	2.19
**NP3**	3.03 (0.02)	7	0.21 (0.04)	95.8 (2.3)	2.96
**NP3/6**	2.87 (0.03)	6	0.24 (0.05)	93.1 (3.0)	2.10
**NP4**	3.23 (0.06)	5	0.53 (0.05)	95.5 (2.7)	3.12
**NP4/6**	3.14 (0.03)	6	0.50 (0.06)	95.0 (2.4)	2.26
**NP5**	3.21(0.03)	-	0.14 (0.04)	87.7 (2.5)	2.85
**NP5/6**	2.96 (0.04)	-	0.29 (0.05)	86.7 (3.6)	2.28

[a]Ligands are assigned to the same bundle based on their relative orientation and end group distances; the (HBDSCAN)[1] algorithm was used to identify sets of ligands that belong to the same bundle. We assigned a minimum number of 4 ligands to form a bundle, which corresponds to the minimum polygon size identified in the Voronoi tessellation.

[b]The asphericity δ gives an indication of shape and is defined as I_z-$(I_x+I_y)/2$, having assigned the principal moments of the gyration tensor as $I_z \geq I_y \geq I_x$. Values close to 0 indicate a spherical form, while values around 1 an oblong shape (e.g., ellipsoid).

[c]The percentage of *trans* dihedrals angles ($-180° < \varphi < -120°$ and $120° < \varphi < 180°$) relative to the total number of dihedral angles in the ligand chain is a measure of ligand ordering. The dihedral angles were calculated taking into account all the heavy atoms of the alkyl portion and ignoring all the hydrogen atoms.

[d]The area dispersion index (ADI) is calculated from the Voronoi tessellation of the ligands center of mass. See **Section S3.3** for ADI definition.

Table S2. Organic overlayer thickness (δ) for selected systems from MD calculations and comparison with the values obtained through XPS measurements.

	δ_{MD} (nm)	δ^a_{XPS} (nm)
**NP1/6**	1.57	1.65
**NP2**	1.90	1.91
**NP2/6**	1.88	1.79
**NP5**	1.58	1.37
**NP5/6**	1.64	1.72

[a]The relative error on the thickness was estimated to be 10%, according to the outcomes described by Shard for NPs with this size.[2]

The thickness of the organic overlayer obtained through XPS measurements are comparable with the value obtained via MD simulations. This supports the molecular models adopted. At the same time, since XPS measurements are carried out on dry samples under high vacuum, it suggests high gold passivation and overall monolayer compactness.

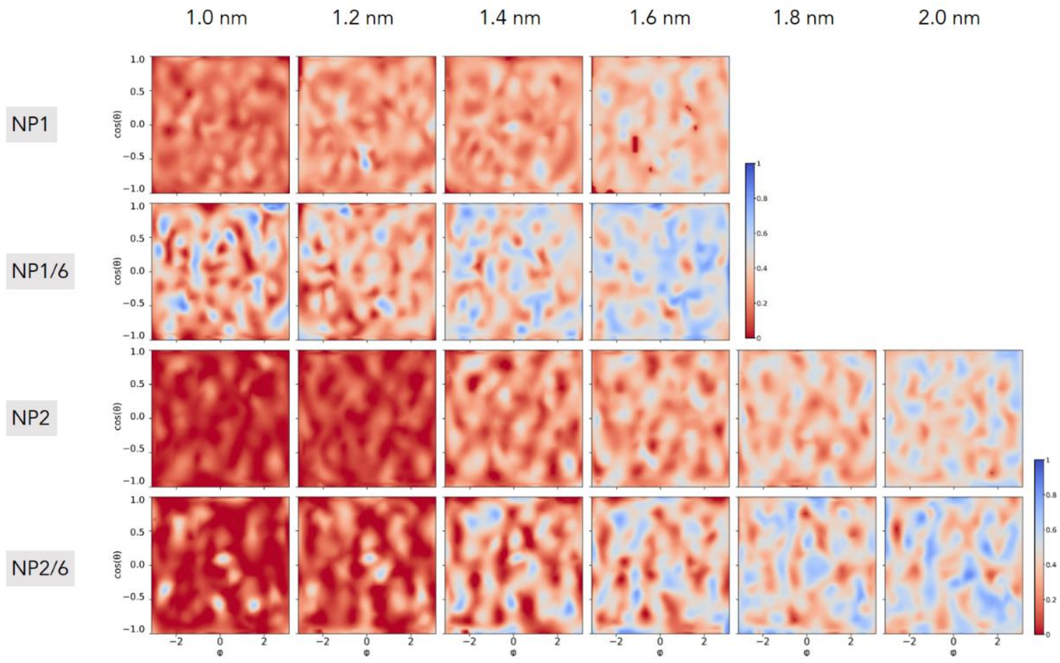

Figure S5. Normalized water distribution at increasing distance from the gold surface for **NP1** and **NP2** series. Color code same as in **Figure 4**.

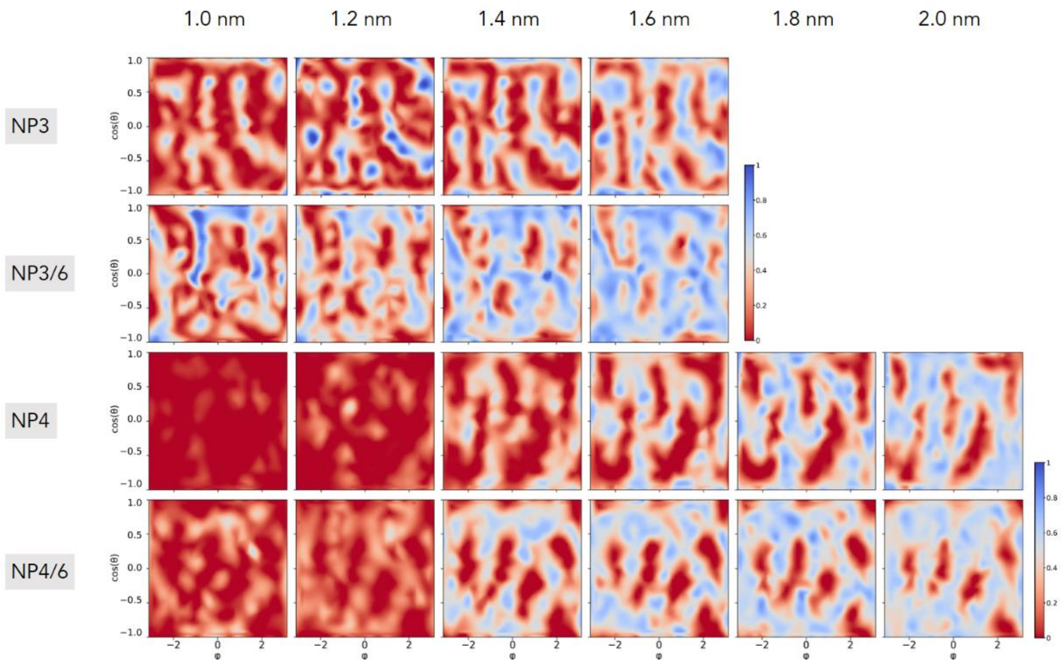

Figure S6. Normalized water distribution at increasing distance from the gold surface for **NP3** and **NP4** series. Color code same as in **Figure 4**.

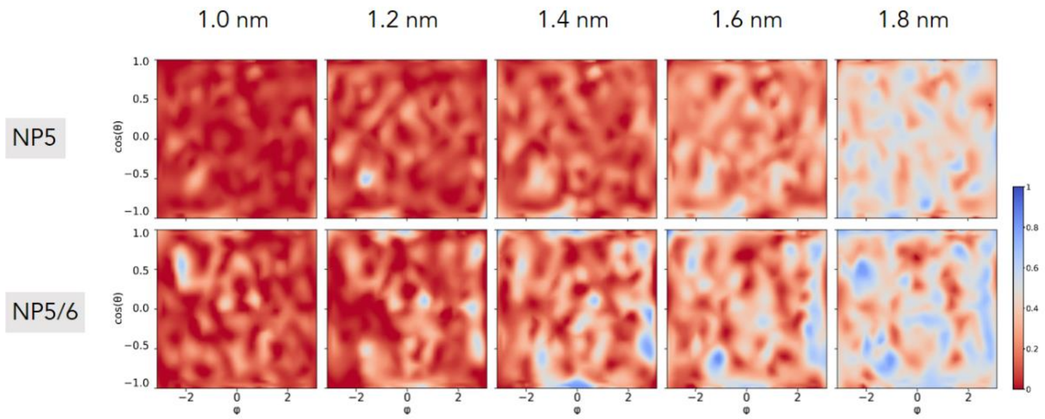

Figure S7. Normalized water distribution at increasing distance from the gold surface for **NP5** serie. Color code same as in **Figure 4**.

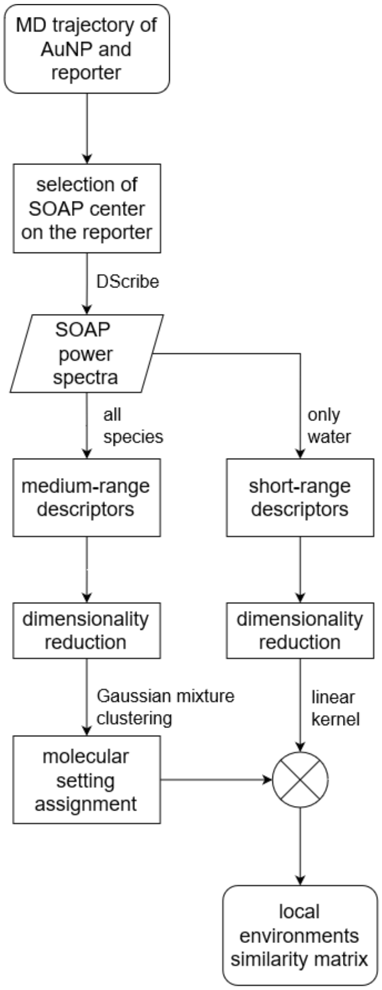

Figure S8. Flowchart of the computational protocol proposed for the identification of local environments within SAMs. Details and parameters for each step are provided in **Section S3** of the Supporting Information.

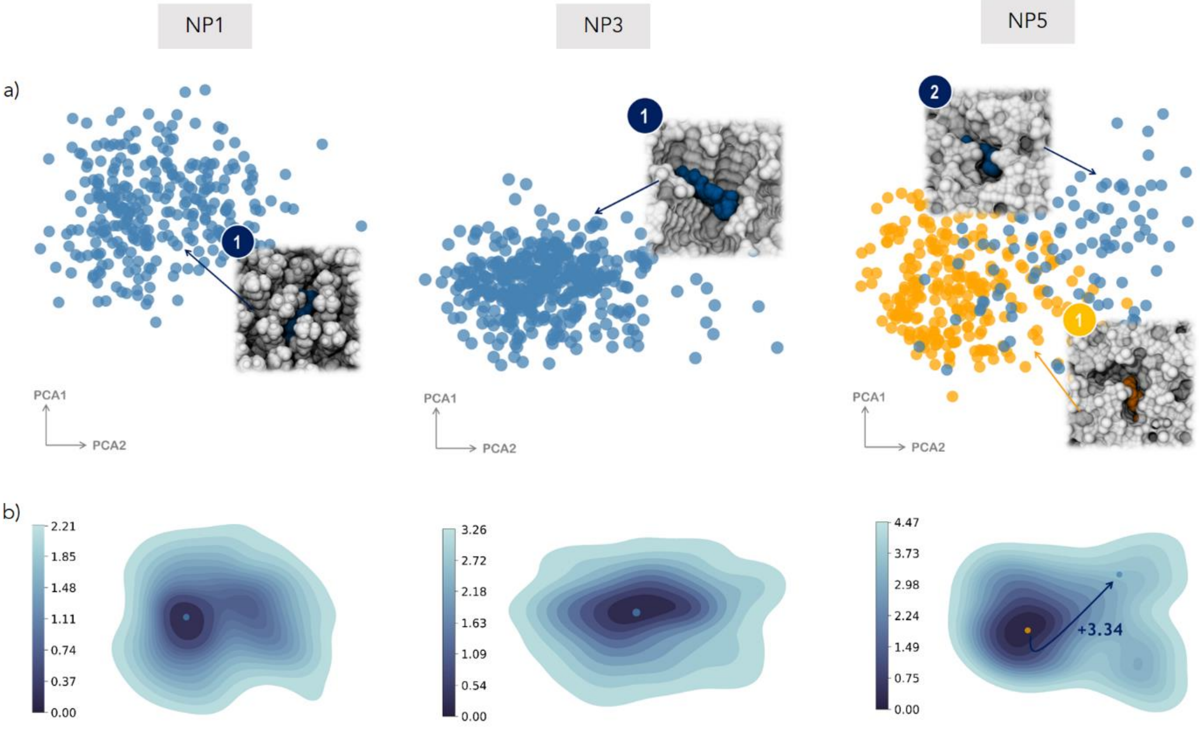

Figure S9. (a) First two principal components (PCA1 and PCA2) obtained from dimensionality reduction of medium-range SOAP feature space of the probe **7** in **NP1**, **NP3**, and **NP5**. Dots are colored according to the clusterization obtained by the GMM analysis. For each cluster, the insert shows a zoomed view of the molecular environment centered on the probe, as extracted from the corresponding MD frames. Water is not shown for clarity. Color legend: probe, same color of the cluster; ligands colored in grey. (b) Free energy surface (FES)(kcal/mol) calculated from the states probability distribution for the same systems in (a). Dots superimposed on the surface identify the clusters and are colored based on the microstate they refer to (panel (a)). The arrow indicates the transition probability from the minimum.

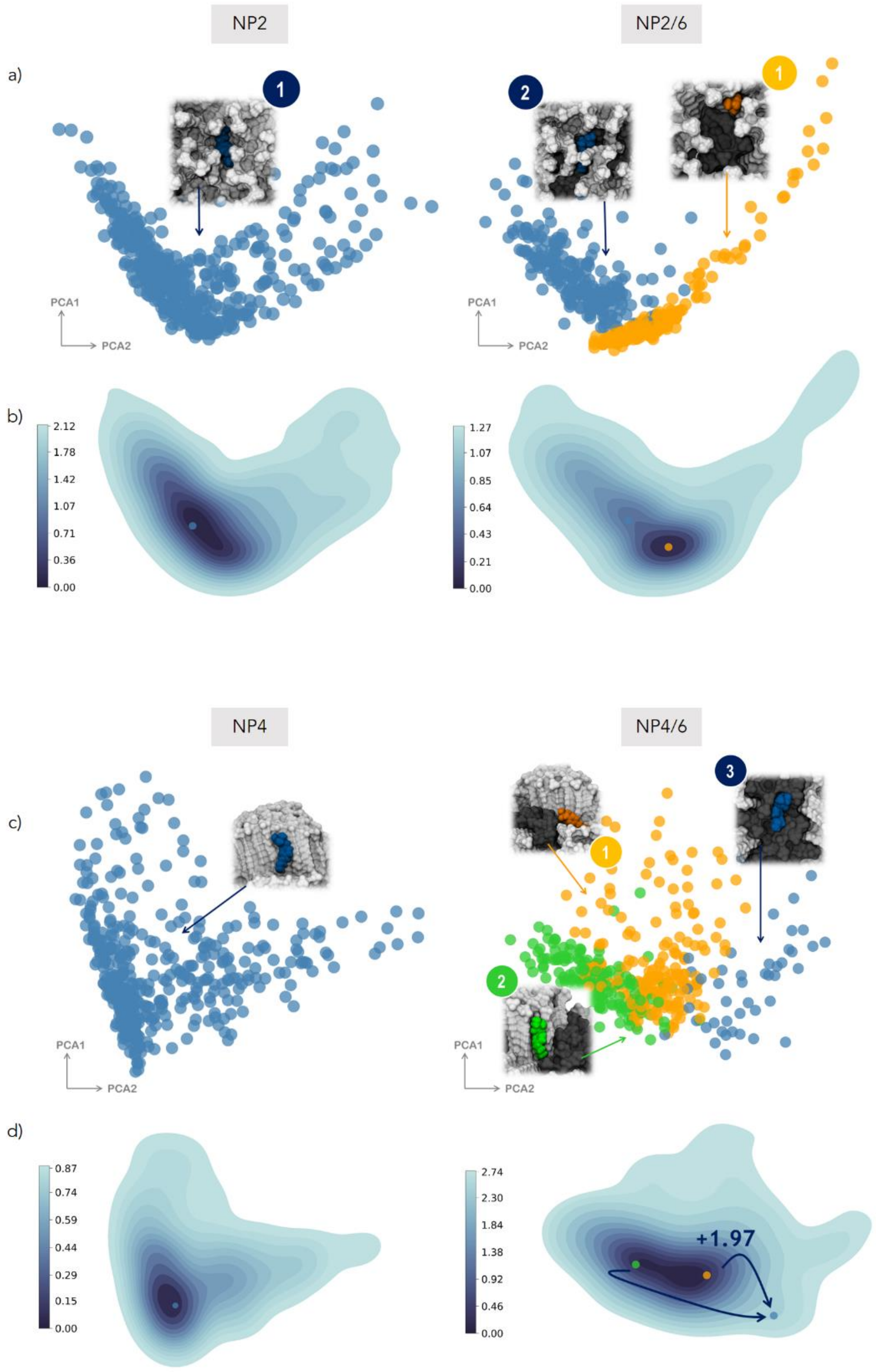

Figure S10. (a) First two principal components (PCA1 and PCA2) obtained from dimensionality reduction of medium-range SOAP feature space of the probe **7** in **NP2**, **NP2/6**, **NP4**, and **NP4/6** at 340 K. Dots are colored according to the clusterization obtained by the GMM analysis. For each cluster, the insert shows a zoomed view of the molecular environment centered on the probe, as extracted from the corresponding MD frames. Water is not shown for clarity. Color legend: probe, same color of the cluster; ligands **2** and **4** colored in grey; ligand **6** colored in dark grey. (b) Free energy surface (FES)(kcal/mol) calculated from the states probability distribution for the same systems in (a). Dots superimposed on the surface identify the clusters and are colored based on the microstate they refer to (panel (a)). The arrow indicates the transition probability from the minimum.

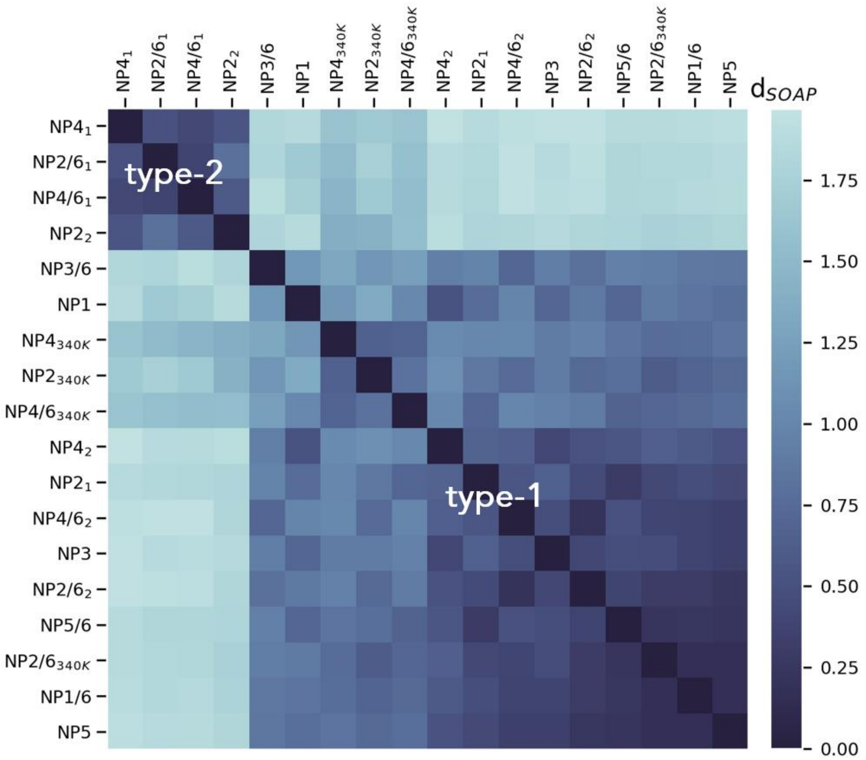

Figure S11. Similarity matrix for all local most visited environments generated by calculating the pairwise SOAP kernels K_{SOAP} between all the reduced short-range SOAP feature vectors. The matrix contains also the states found at high temperature (340 K). The latter ones are marked explicitly. Dark blue color indicates high similarity between the environments.

S2. SYNTHESIS AND CHARACTERIZATION OF MIXED-MONOLAYERS NANOPARTICLES

S2.1. Synthesis of NP-C12N/F6 (NP1/6)

To a solution of tetrachloroauric acid (100 mg, 0.296 mmol) in 11.6 mL of deoxygenated milliQ water, TOAB (869 mg, 1.59 mmol) in 8.8 mL of deoxygenated chloroform was added and the solution was let to stir for 30 min at room temperature. The two phases were separated and sodium borohydride, in 7.8 mL milliQ water, was added (161.4 mg, 4.27 mmol) to the organic phase and stirred for 15 minutes under argon atmosphere. After this time, the mixture of the two thiols was added (1.8 mg HS-F6 and 8.6 mg HS-C12N) in 6 mL isopropanol and the nanoparticles precipitated. After 1.3 h the solid was separated and the nanoparticles were washed six times with chloroform (6 x 15 mL) (4500 rpm, 4 min, 25 °C). After decomposition of the NPs using a solution of I_2 in methanol, a 2.1/1 ratio HS-C12N/F6 was found from the integrals of the ^{1}H NMR spectrum. TEM: 4.5 ± 1.0 nm, TGA 15 %, $Au_{3100}C12N_{245}F_{117}$.

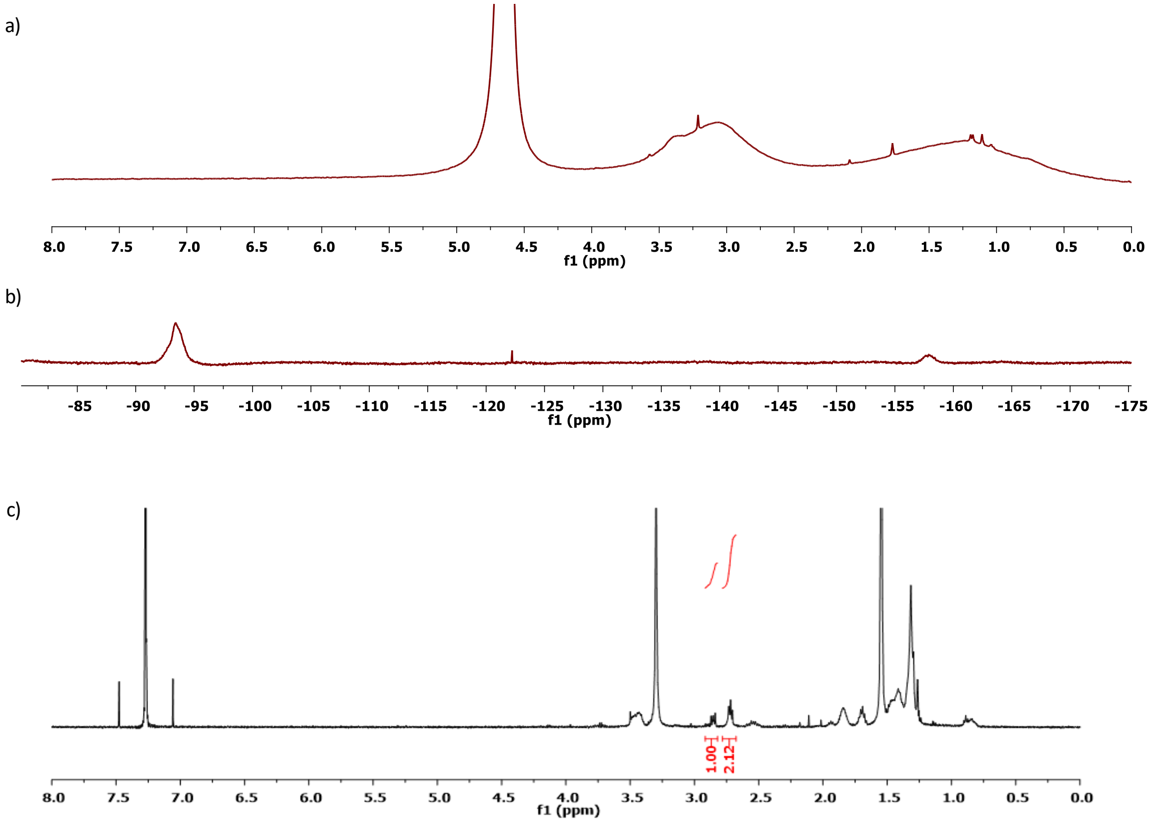

Figure S12. a) ^{1}H-NMR (400 MHz, D_2O) spectra; b) ^{19}F NMR (376.16 MHz, D_2O) spectra of NP-C12N/F6 and ^{1}H NMR (400 MHz, $CDCl_3$) of decomposed NP-C_{12}N/F_6 with integrals of the signals at: 2.92 – 2.82 ppm assigned to S-S-<u>CH$_2$</u>-CH$_2$-(CF$_2$)$_5$-CF$_3$ and at 2.70 – 2.75 ppm assigned to S-S-CH$_2$-CH$_2$-(CH$_2$)$_{10}$-N$^+$(CH$_3$)$_3$.

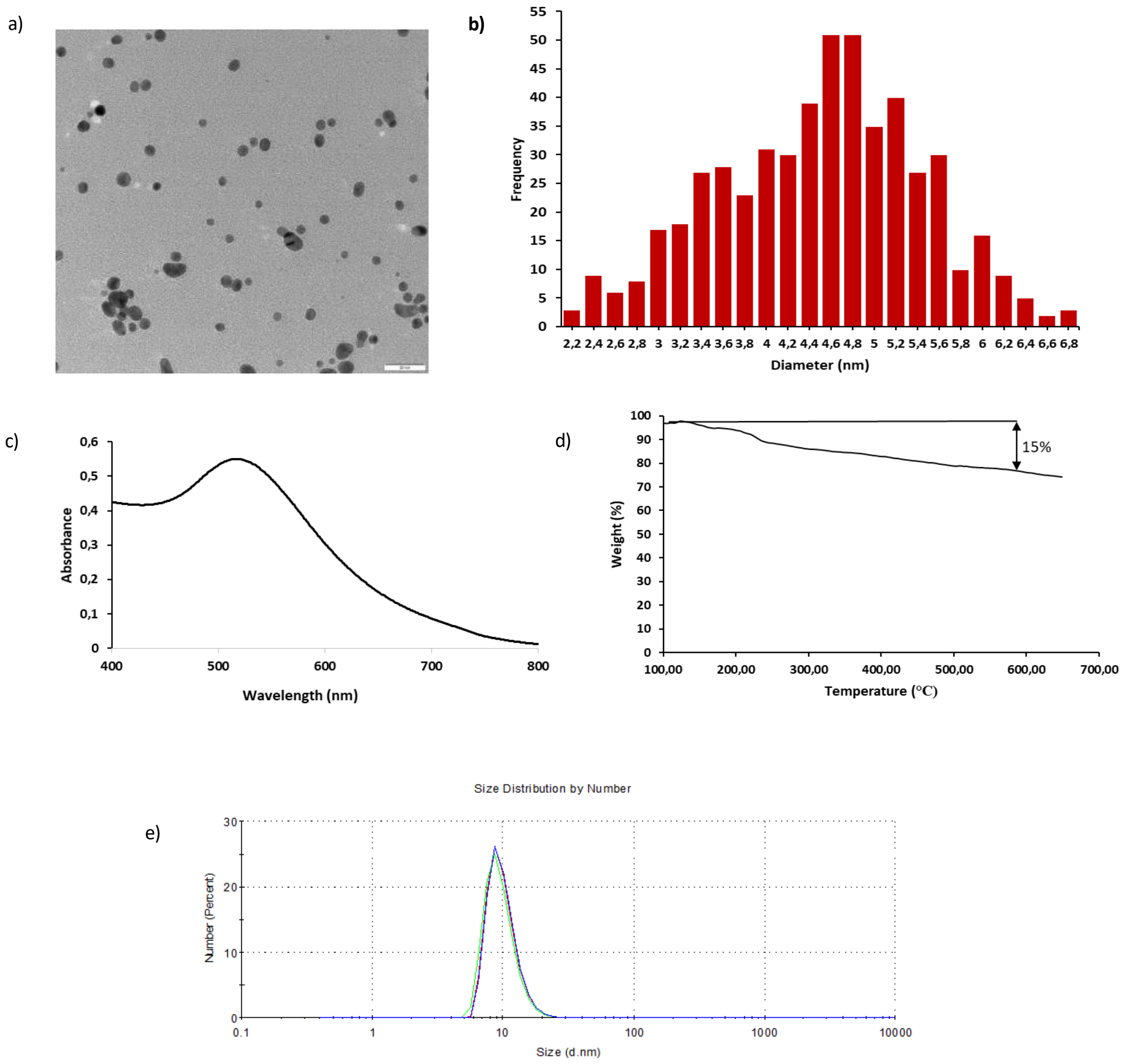

Figure S13. a) Representative TEM image (scale bar: 200 nm); b) Size histogram; c) UV-VIS (H_2O, 0,1 mg/mL); d) TGA analysis and e) hydrodynamic diameter size distribution 9.9 ± 2.6 nm, n = 3, determined by DLS of NP-C12N/F6.

S2.2. Synthesis of NP-C16N/F6 (NP2/6)

To a solution of tetrachloroauric acid (206.7 mg, 0.608 mmol) in 24.7 mL of deoxygenated milliQ water, TOAB (1.796 g, 3.28 mmol) in 18.6 mL of deoxygenated chloroform was added and the solution was let to stir at room temperature for 30 min. The two phases were separated and sodium borohydride (330 mg, 8.81 mmol in 16.8 mL milliQ water) was added to the organic phase and stirred for 15 minutes under argon atmosphere. After this time, the mixture of the two thiols was added (20 mg HS-F6 and 5.3 mg HS-C16N) in 16.8 mL of isopropanol and the nanoparticles precipitated. After 2 h the solid was separated and the nanoparticles were

washed six times with chloroform (6 x 15 mL) (4500 rpm, 4 min, 25 C). After the decomposition of the monolayer, a 3.7/1 ratio HS-C16N/F6 was found. To a solution of these nanoparticles (60 mg dissolved in 30 mL deoxygenated MeOH), 1 mg of HS-F6 was added and the mixture was let to stir at 40 °C for 3 days. After decomposition of the NPs using a solution of I_2 in methanol, 2.3/1 ratio HS-C16N/F6 was found from the integrals of the ^{1}H NMR spectrum. TEM: 4.1 ± 0.9 nm, TGA 18.4 %, $Au_{2600}C16N_{237}F_{103}$.

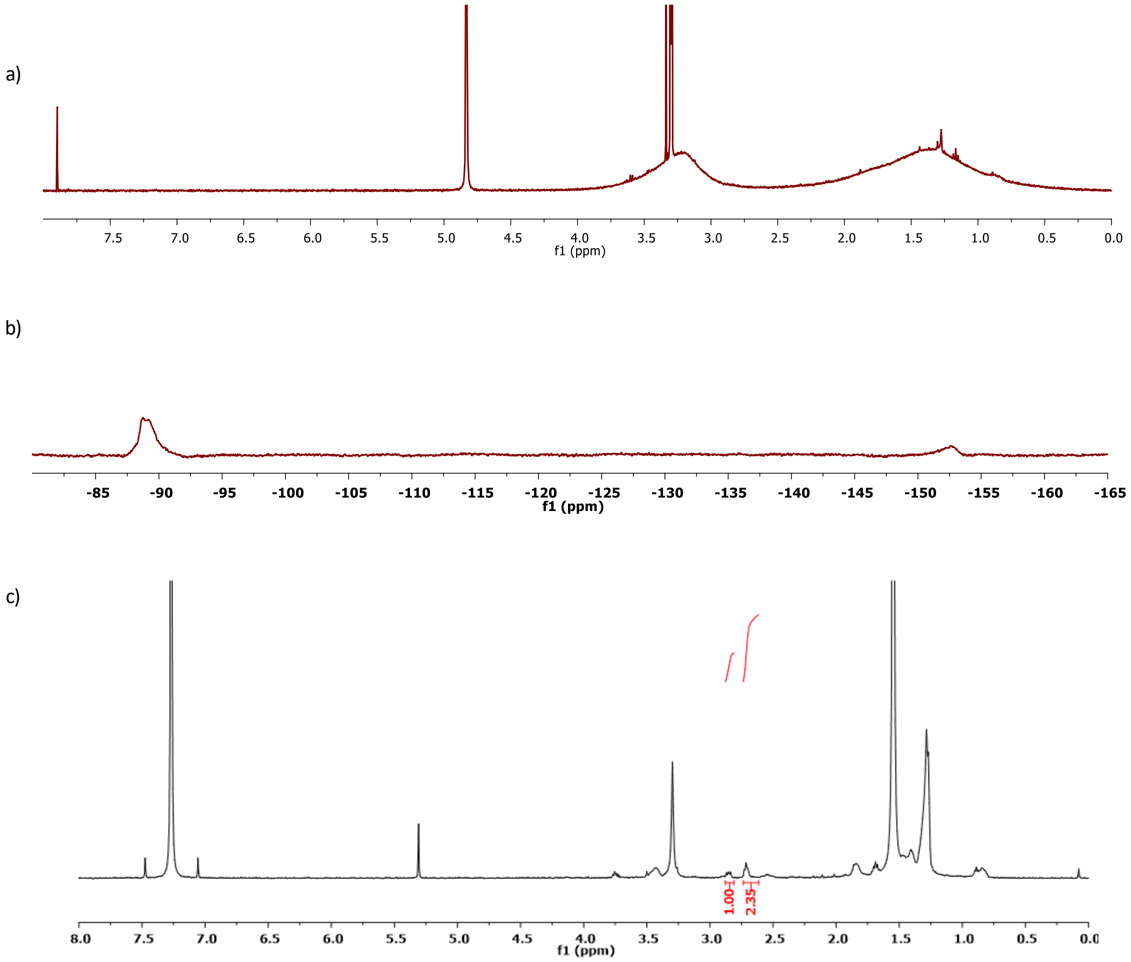

Figure S14. a) ^{1}H-NMR (400 MHz, D_2O); b) ^{19}F NMR (376.16 MHz, D_2O) spectra of NP-C16N/F6 and c) ^{1}H NMR (400 MHz, $CDCl_3$) of decomposed NP-$C_{16}N/F_6$ with integrals of the signals at: 2.87 – 2.82 ppm for S-S-CH_2-CH_2-$(CF_2)_5$-CF_3 and at 2.68 – 2.75 ppm: S-S-CH_2-CH_2-$(CH_2)_{14}$-$N^+(CH_3)_3$.

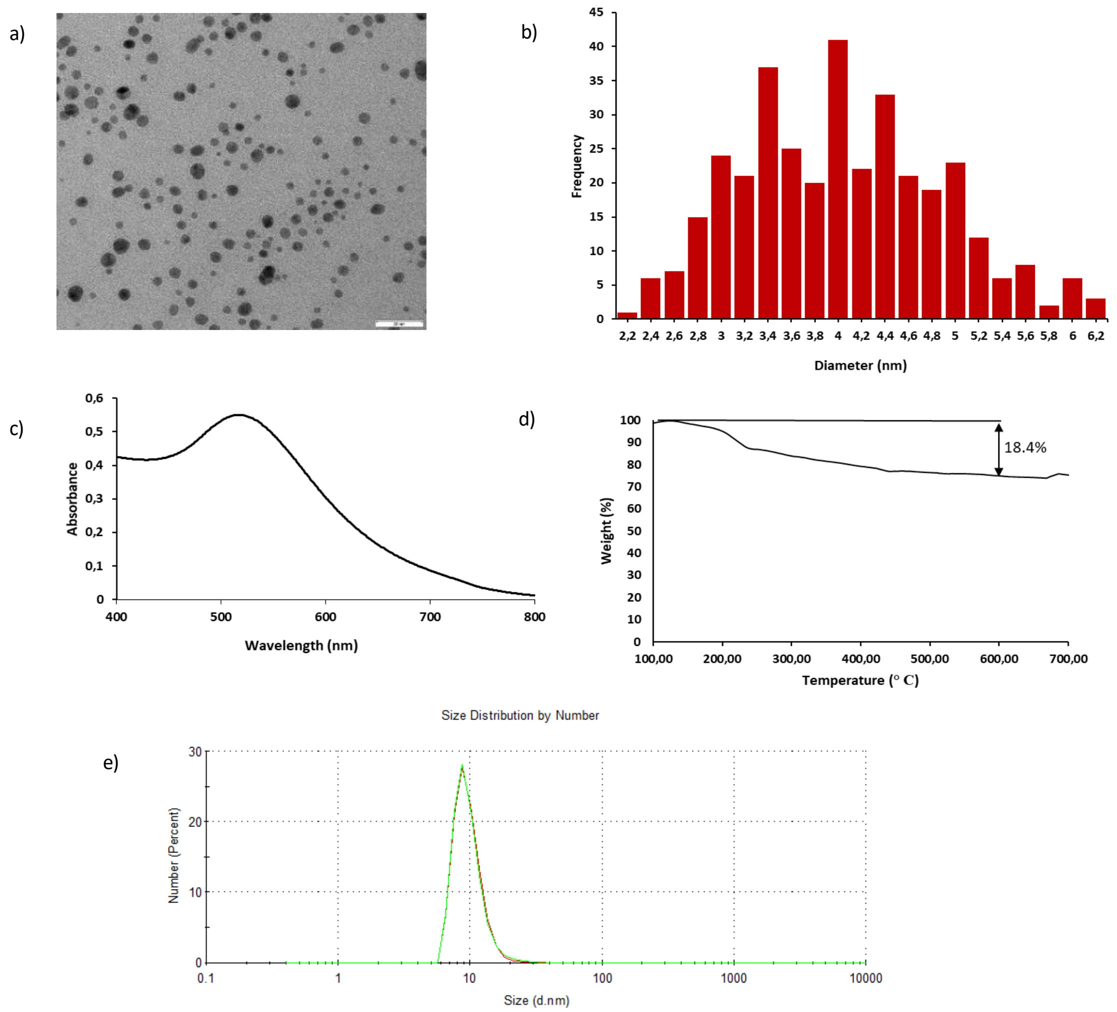

Figure S15. a) Representative TEM image (scale bar: 200 nm); b) Size histogram; c) UV-VIS (H_2O, 0,1 mg/mL); d) TGA analysis and e) hydrodynamic diameter size distribution 9.7 ± 2.8 nm, n = 2, determined by DLS of NP-C16N/F6.

S2.3. Synthesis of NP-ZWPN/F6 (NP5/6)

A solution of TOAB (1.3 g, 5.4 Eq) in chloroform (13.5 mL) was added under argon atmosphere to an aqueous solution of tetrachloroauric acid (0.15 g, 1 Eq in 17.9 mL) at 25 °C and the reaction was let to stir for 15 minutes. The two phases were separated and a solution of sodium borohydride (0.241 g, 14.5 Eq) in water (12.2 mL) was added to the organic phase. The red colored solution was stirred for 15 minutes and the mixture of the thiols was added (13.3 mg ZW-PN (36.1 mmol) and 13.7 mg F6 (36.1 mmol) in 12.2 mL isopropanol. The nanoparticles precipitated and the dispersion was stirred for 2 hours. The solid was separated and washed five times with chloroform (30 mL, 4500 rpm, 5 min). The as-formed nanoparticles have been decomposed with a solution of I_2 in methanol in order to obtain the ratio between the two thiols present into the monolayer and

the nanoparticles were analyzed on TEM to determine the dimension of the gold core. ZW-PN/F6 = 2.5/1. TEM: 4.2 ± 0.9 nm, TGA 19 % (Au_{2750}ZW-$PN_{244}F_{98}$).

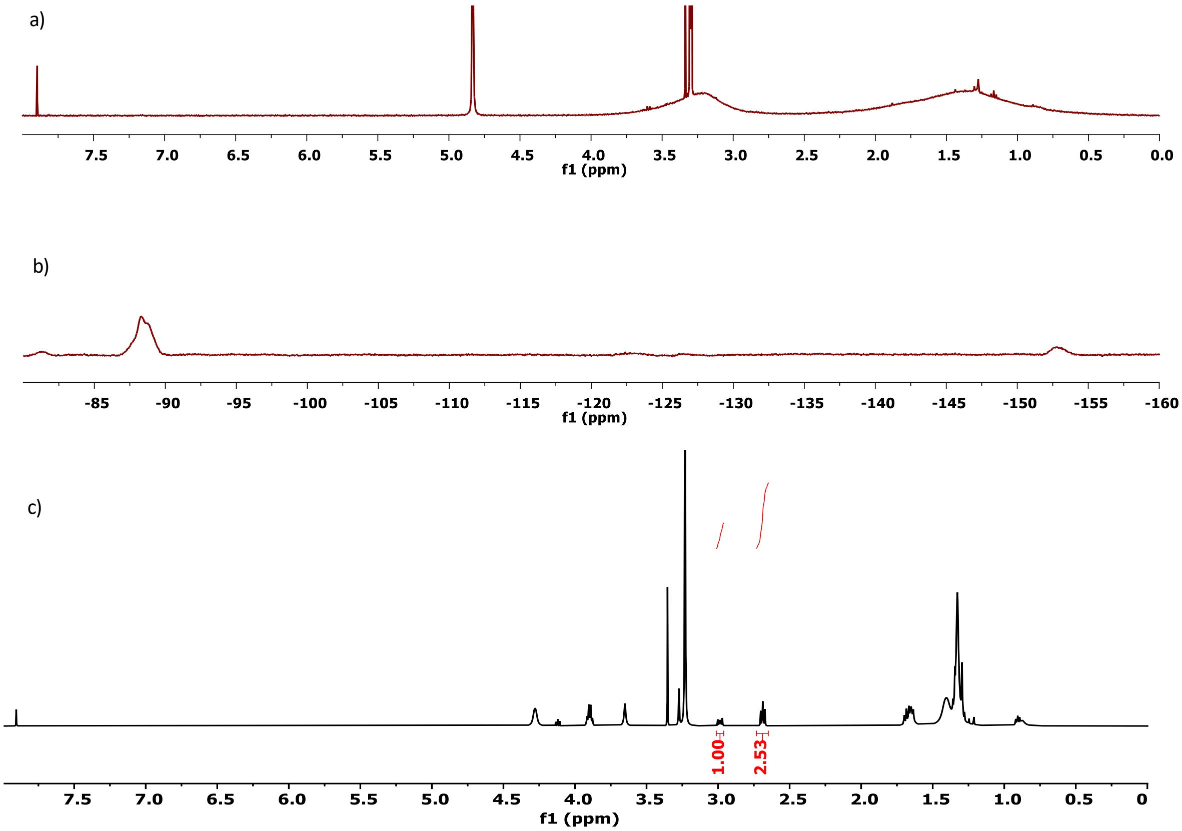

Figure S16. a) ^{1}H-NMR (400 MHz, D_2O); b) ^{19}F NMR (376.16 MHz, D_2O) spectra of NP-ZWPN/F6 and c) ^{1}H NMR (400 MHz, CD_3OD) of decomposed NP-ZWPN/F6 with integrals of the signals at 3.00 – 2.95 ppm assigned to S-S-CH_2-CH_2-$(CF_2)_5$-CF_3 and at 2.70 – 2.68 ppm assigned to S-S-CH_2-CH_2-ZWPN.

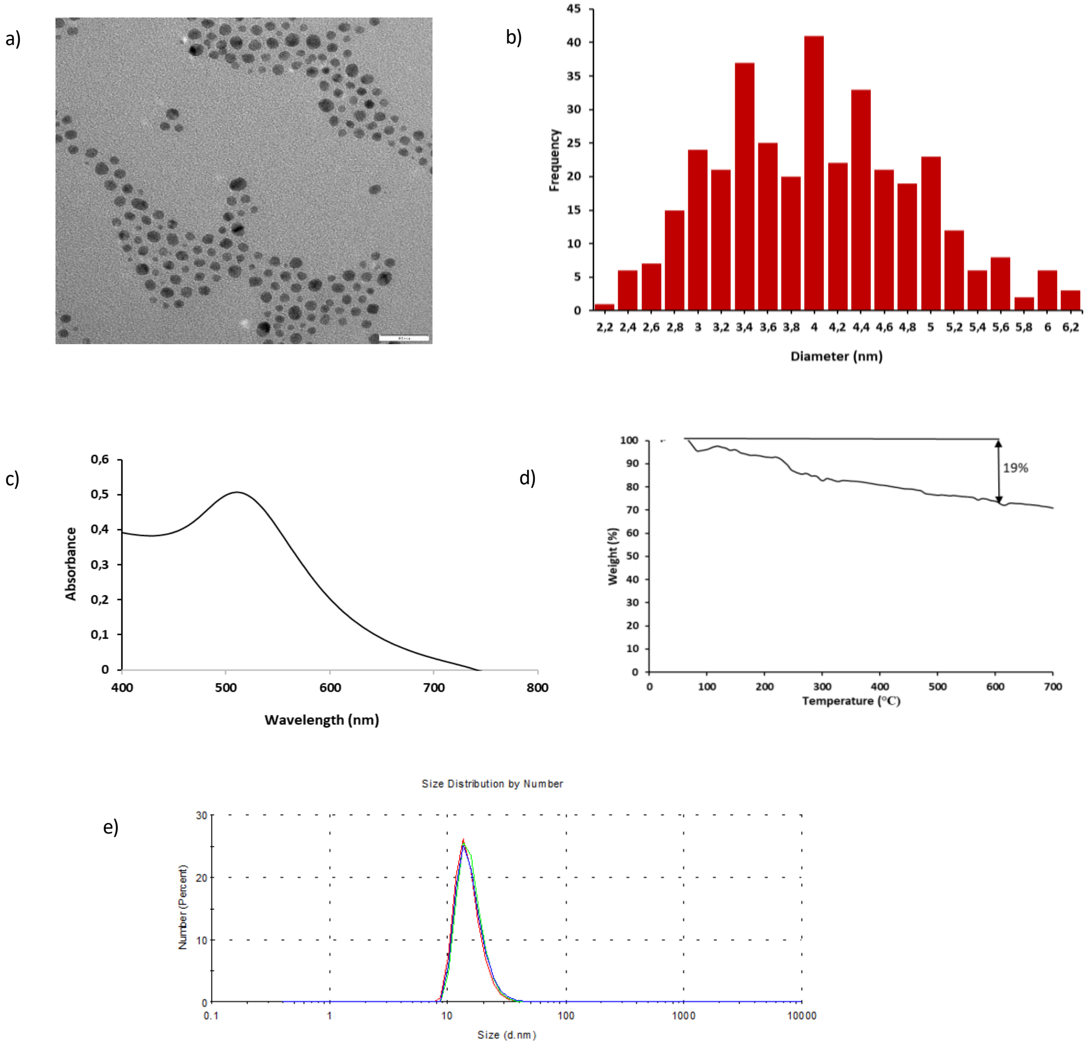

Figure S17. a) Representative TEM image (scale bar: 200 nm); b) Size histogram; c) UV-VIS (H_2O, 0,1 mg/mL); d) TGA analysis and e) hydrodynamic diameter size distribution 15.7 ± 4.6 nm, n = 3, determined by DLS of NP-ZWPN/F6.

S2.4. Synthesis NP-MDDS/F6 (NP3/6)

$HAuCl_4 \cdot 3H_2O$ (50 mg, 0.147 mmol, 1 eq) was dissolved in 5.8 mL of deoxygenated water and stirred for 30 min at 25°C with a solution of TOABr (434.5 mg, 0.795 mmol, 5.4 Eq) in 4.4 mL of deoxygenated chloroform pre-treated with potassium carbonate. After removal of the colorless aqueous layer, a cold solution of $NaBH_4$ (80.7 mg, 2.133 mmol, 14.5 Eq) in 3.9 mL deoxygenated water was quickly added to the orange organic phase containing gold and the mixture was stirred vigorously for 15 min at 25°C. Then the aqueous layer was removed and a dark red-violet solution of nanoparticles in chloroform was obtained. MDDS 4.2 mg (0.014 mmol) was dissolved in 2 mL of 1:1 deoxygenated methanol: isopropanol mixture, while HS-F6 1.75 mg

(0.0046 mmol) was dissolved in 1 mL of deoxygenated methanol. The thiols solutions were joined to obtain a clear 3/1 MDDS/F6 solution that was added dropwise to the nanoparticle's solution. After stirring for 1.2 h at 25°C, nanoparticles were precipitated by centrifugation (4200 rpm for 5 min) and washed with chloroform pretreated with K_2CO_3 (5x20 mL) and methanol (5x20 mL). Nanoparticles were further purified by G-75 Sephadex chromatography in water.

The obtained nanoparticles were characterized by ^{1}H-NMR spectroscopy, TEM and UV-vis spectroscopy. After decomposition of the NPs using a solution of I_2 in methanol, a 2.1/1 ratio HS-MDDS/F6 was found from the integrals of the ^{1}H NMR spectrum. TEM: 4.5 ± 0.9 nm, TGA 15 %, $Au_{2975}MDDS_{239}F_{132}$.

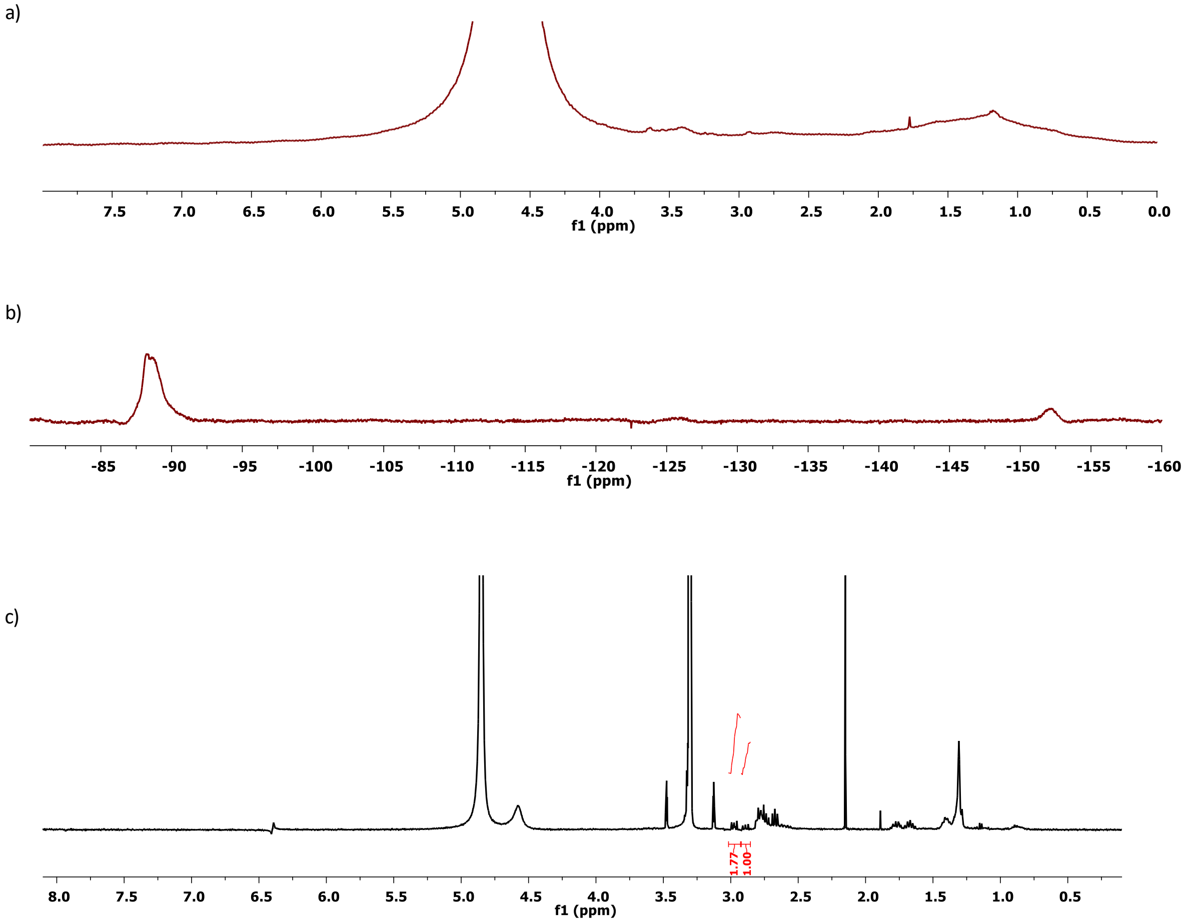

Figure S18. a) ^{1}H-NMR (400 MHz, D_2O); b) ^{19}F NMR (376.16 MHz, D_2O) spectra of NP-MDDS/F6 and c) ^{1}H NMR (400 MHz, CD_3OD) of decomposed NP-MDDS/F_6: integrals of the signals at: 3.00 – 2.95 ppm assigned to S-S-CH_2-CH_2-$(CF_2)_5$-CF_3 and at 2.92 – 2.85 ppm assigned to S-S-CH_2-$(CH_2)_{10}$-CH_2-SO_3^-.

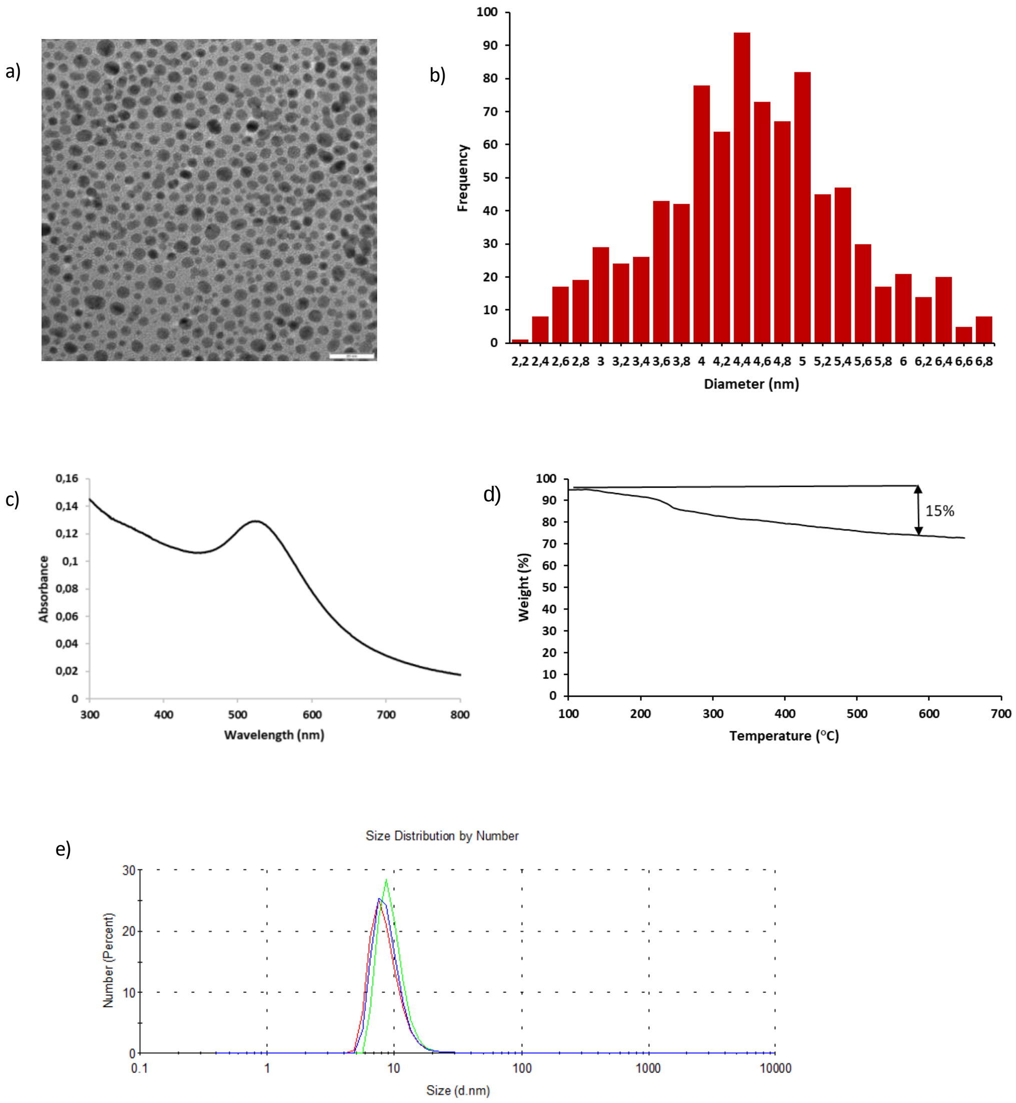

Figure S19. a) Representative TEM image (scale bar: 200 nm); b) Size histogram; c) UV-VIS (H_2O, 0,1 mg/mL); d) TGA analysis and e) hydrodynamic diameter size distribution 8.9 ± 2.6 nm, n = 3, determined by DLS of NP-MDDS/F6.

S2.5. Synthesis NP-MHDS/F6 (NP4/6)

$HAuCl_4{\cdot}3H_2O$ (50 mg, 0.147 mmol, 1 eq) was dissolved in 5.8 mL of deoxygenated water and stirred for 30 min at 25 °C with a solution of TOABr (434.5 mg, 0.795 mmol, 5.4 eq) in 4.4 mL of deoxygenated chloroform pretreated with potassium carbonate. After the colorless aqueous layer was discarded and a cold solution of $NaBH_4$ (80.7 mg, 2.133 mmol, 14.5 eq) in 3.9 mL deoxygenated water was quickly added to the orange organic phase containing gold and the mixture was vigorously stirred for 15 min at 25°C. Finally, the aqueous layer was removed and a dark red-violet solution of nanoparticles in chloroform was obtained. A solution of MHDS and F6 in a 5/1 ratio was prepared. 5.4 mg (0.015 mmol) of MHDS were dissolved in a 2.3 mL of 1:1:0.3

deoxygenated methanol:isopropanol:DMF mixture, while F6 (1.14 mg, 0.003 mmol) was dissolved in 1 mL of deoxygenated methanol. Finally, the two thiols solutions were joined to give a clear MHDS/F6 solution that was added dropwise to the nanoparticle's solution. After stirring for 1.20 h at 25°C, nanoparticles were precipitated by centrifugation (4200rpm for 5 min) and washed with chloroform pretreated with K_2CO_3 (5x20 mL) and methanol (5x20 mL). Nanoparticles were further purified by G-75 Sephadex chromatography performed in water. The obtained nanoparticles were characterized by ^{1}H-NMR spectroscopy, TEM and UV-vis spectroscopy. After decomposition of the NPs using a solution of I_2 in methanol, a 2.2/1 ratio HS-MHDS/F6 was found from the integrals of the ^{1}H NMR spectrum. TEM: 4.5 ± 0.8 nm, TGA 15 %, $Au_{3100}MHDS_{275}F_{125}$.

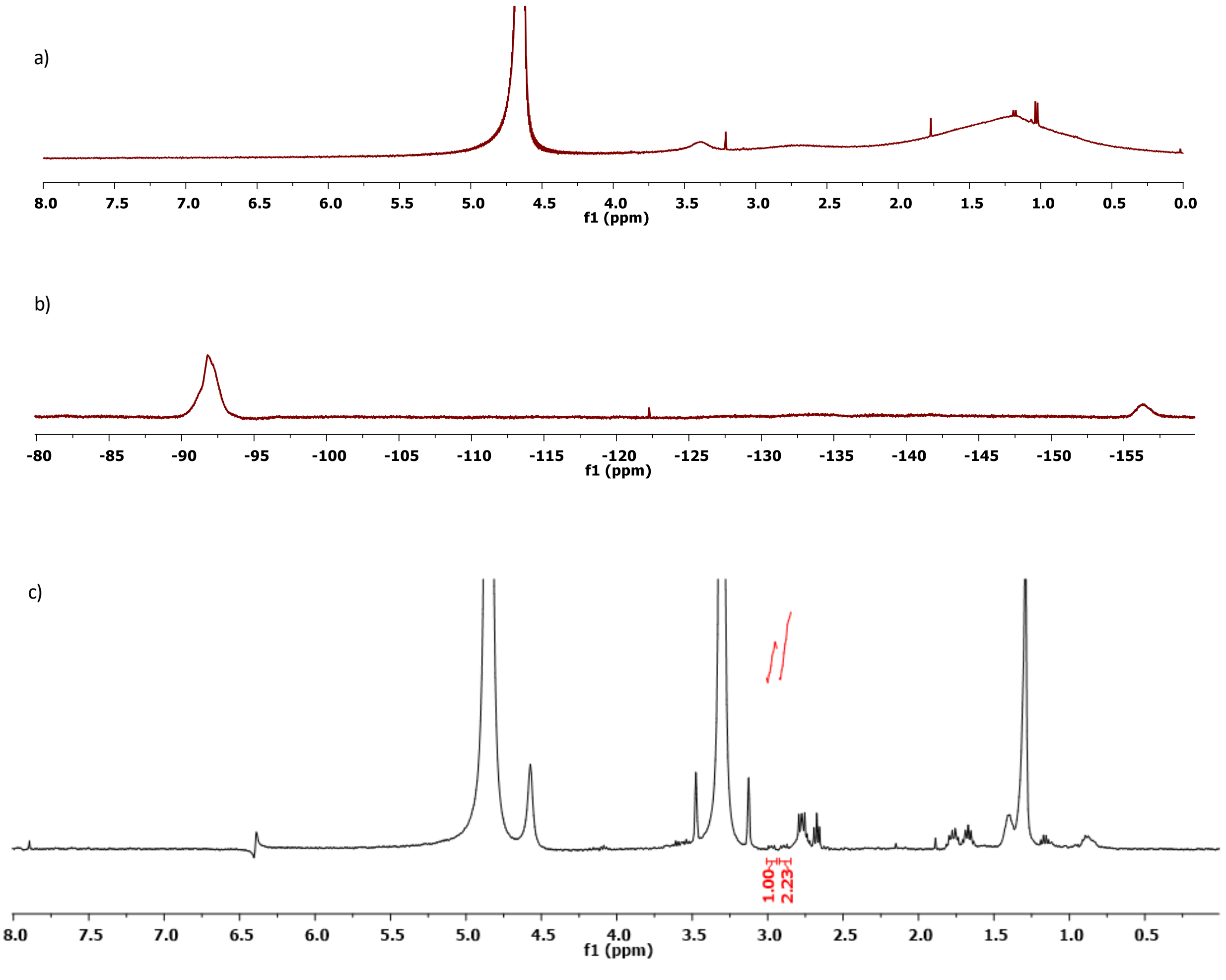

Figure S20. a) ^{1}H-NMR (400 MHz, D_2O), b) ^{19}F NMR (376.16 MHz, D_2O) spectra of NP-MHDS/F6 and c) ^{1}H NMR (400 MHz, CD_3OD) of decomposed NP-MHDS/F6: integrals of the signals at: 3.00 – 2.95 ppm assigned to S-S-CH_2-CH_2-$(CF_2)_5$-CF_3 and at 2.92 – 2.87 ppm assigned to S-S-CH_2-$(CH_2)_{14}$-CH_2-SO_3^-.

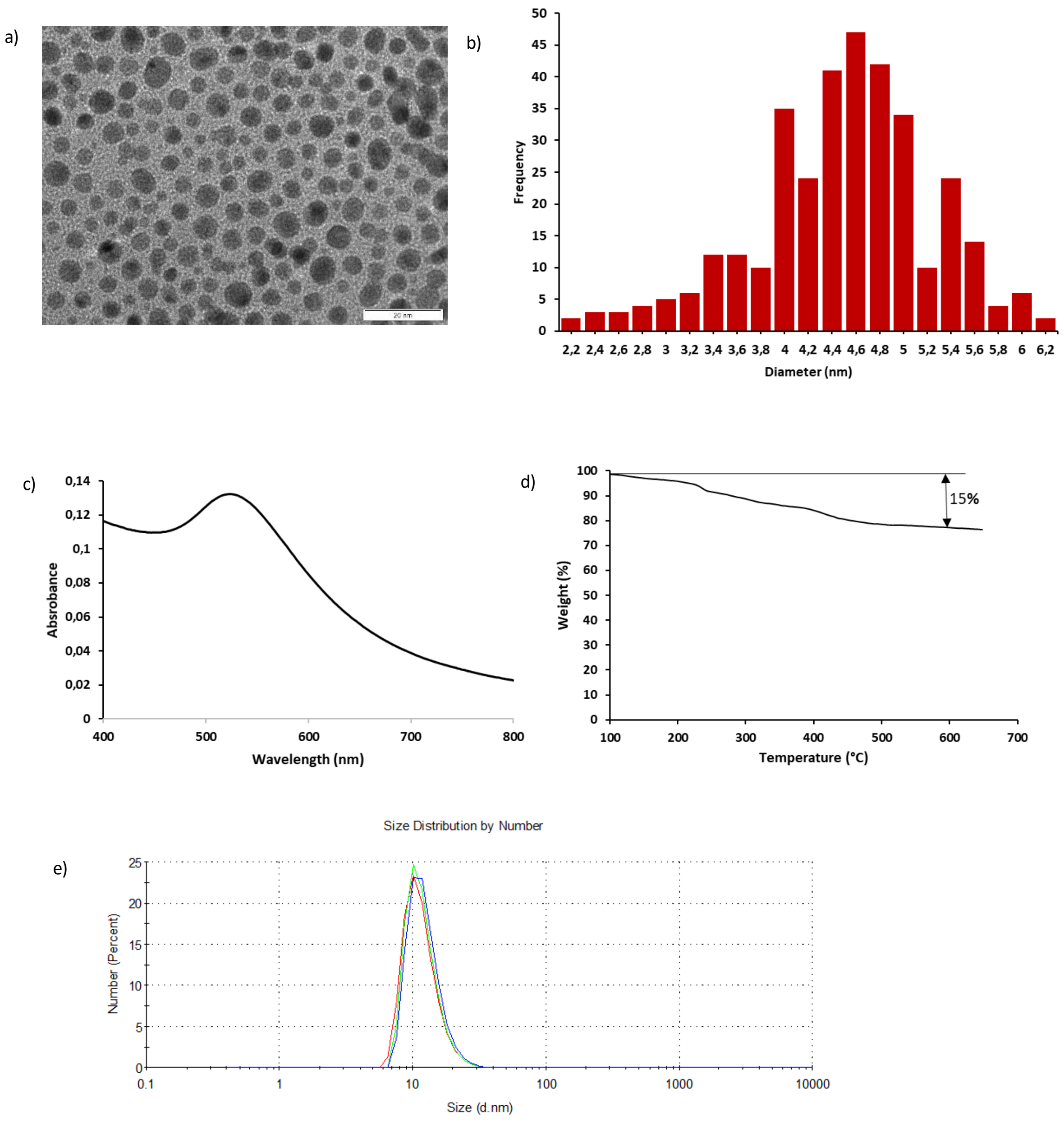

Figure S21. a) Representative TEM image (scale bar: 200 nm); b) Size histogram; c) UV-VIS (H_2O, 0,1 mg/mL); d) TGA analysis and e) hydrodynamic diameter size distribution 12.3 ± 3.6 nm, n = 3, determined by DLS of NP-MHDS/F6.

S3. COMPUTATIONAL METHODS

S3.1. Dissipative particle dynamics (DPD)

Theory. Dissipative particle dynamics (DPD)[3] is a well-established mesoscale stochastic simulation method using coarse-grained particles. In DPD simulations, a small group of atoms or molecules are lumped (i.e. coarse-grained) into a single unit (DPD bead) and the dynamics of each bead is governed by Newton's equation of motion. Thanks to this lumping, DPD gives rise to decreased degrees of freedom, which enables one to analyze complex systems at a bigger time and length scales, thus making it computationally efficient and cheaper. Accordingly, DPD resembles molecular dynamics, but with this technique, it is possible to analyze complex phenomena such as self-assembly of the amphiphilic molecules, phase separations, among others, which are not feasible with classical methods due to limited time and length scales.[4–9]

In DPD each bead *i* is described by position $\boldsymbol{r}_i$, velocity $\boldsymbol{v}_i$, mass m_i and interact with other beads by the force $\boldsymbol{F}_i$ written as a sum of three pairwise forces and additional forces that reflect bonds, bending, etc. in complex molecules, active within an interaction range defined by the cutoff distance r_c:

$$\boldsymbol{F}_i = \sum_{i\neq j} \boldsymbol{f}_{ij}^{C} + \sum_{i\neq j} \boldsymbol{f}_{ij}^{D} + \sum_{i\neq j} \boldsymbol{f}_{ij}^{R} + \sum_{i\neq j} \boldsymbol{f}_{ij}^{bond} + \sum_{i\neq j\neq z} \boldsymbol{f}_{ijz}^{angle}$$

$$\boldsymbol{f}_{ij}^{C}(\mathbf{r}_{ij}, a_{ij}) \quad = a_{ij}\left(1 - \frac{\mathbf{r}_{ij}}{r_c}\right)\hat{\boldsymbol{r}}_{ij}$$

$$\boldsymbol{f}_{ij}^{D}(\mathbf{r}_{ij}, \mathbf{v}_{ij}, \gamma_{ij}) = -\gamma_{ij}\, \omega^{D}(\mathbf{r}_{ij})(\mathbf{r}_{ij}\cdot\mathbf{v}_{ij})\hat{\boldsymbol{r}}_{ij}$$

$$\boldsymbol{f}_{ij}^{R}(\mathrm{r}_{ij}, \sigma_{ij}, \xi_{ij}) = \sigma_{ij}\omega^{R}(\mathbf{r}_{ij})\zeta_{ij}\Delta t^{-1/2}\hat{\boldsymbol{r}}_{ij}$$

where $\boldsymbol{f}_{ij}^{C}(\mathbf{r}_{ij}, a_{ij})$ is a conservative force, $\boldsymbol{f}_{ij}^{D}(\mathbf{r}_{ij}, \mathbf{v}_{ij}, \gamma_{ij})$ a dissipative force and $\boldsymbol{f}_{ij}^{R}(\mathrm{r}_{ij}, \sigma_{ij}, \xi_{ij})$ a random force, $\mathbf{r}_{ij} = \mathbf{r}_i - \mathbf{r}_j$, $\hat{\boldsymbol{r}}_{ij}$ is the unit vector, a_{ij} is the maximum repulsion between two beads, $\mathbf{v}_{ij} = \mathbf{v}_i - \mathbf{v}_j$, Δt is the time step of the integration scheme, γ_{ij} and σ_{ij} are the amplitudes of the dissipative and random force, and ζ_{ij} is a Gaussian random number with zero mean and unit variance, which is chosen independently for each pair of beads. The conservative force $\boldsymbol{f}_{ij}^{C}$ represents the excluded volume interactions between two beads and reflects the chemistry behind the beads; the dissipative $\boldsymbol{f}_{ij}^{D}$ and random force $\boldsymbol{f}_{ij}^{R}$ act as heat sink and source, respectively, and the combined effect of the two forces performs as a thermostat, where the friction coefficient γ_{ij} is related to the thermal noise amplitude σ_{ij} via the fluctuation–dissipation theorem such as

$$\omega^D(\mathbf{r}_{ij}) = [\omega^R(\mathbf{r}_{ij})]^2$$
$$\sigma_{ij}^2 = 2\gamma_{ij} K_B T$$

In addition, our DPD simulations contain also bonds described by a harmonic spring force $\boldsymbol{f}_{ij}^{bond}$, where k_b is the stiffness of the spring and r_0 is the equilibrium distance between the bonded particles *ij*, and a harmonic bond angle force $\boldsymbol{f}_{ijz}^{angle} = \frac{1}{2}K_\theta \sin(\theta - \theta_0)$, where K_θ is the spring constant and θ_0 the equilibrium angle between adiacent bead triples *ijz* in a row.

All the variables are rescaled in DPD simulations by the particle mass *m*, the cutoff distance r_c, and energy unit $k_B T$, where are k_B is the Boltzmann factor and *T* is the absolute temperature.

Models and simulation details. The CG model of the gold (Au) core is an icosahedron of ~ 4 nm size (in rescaled units) made of Au DPD beads arranged on an *fcc* lattice. Each ligand is represented by a flexible chain of DPD beads connected by harmonic springs (**Figure S22**). Water (W) and counterions (CI) (Na+, Cl-) are described as single-bead molecules.

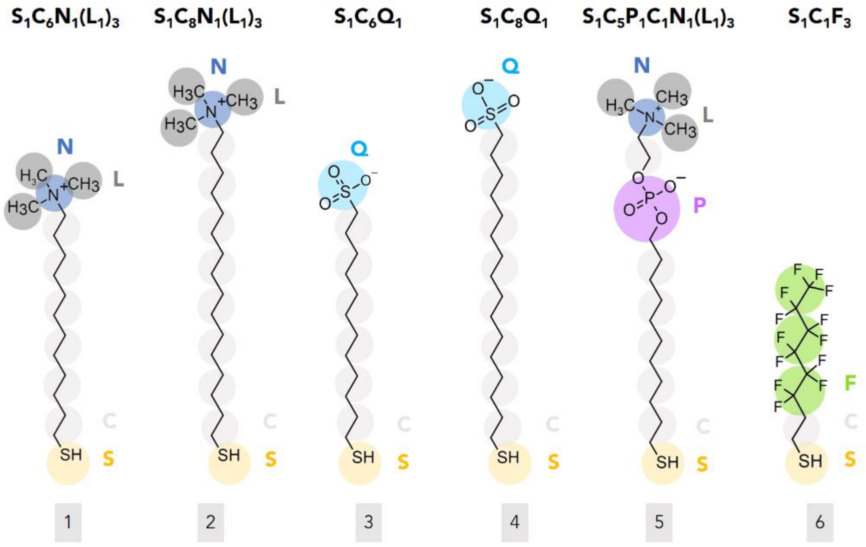

Figure S22. DPD bead-spring model of the ligands installed on mixed SAMs investigated in this study. Each model shows the kind of DPD bead and, on top, the topology of the CG chain, where the subscript indicates the number of each bead type.

The number of ligands was assigned to each nanoparticle to match the experimental number of chain for nm^2. Each ligand was placed close to the gold surface and oriented outward with the head-tail vector along the radial direction. The nanoparticle was then solvated by Packmol,[10] assuming a standard bead density ρ of 3, and placed in the middle of a 3D periodic simulation box of $32r_c$ x $32r_c$ x $32r_c$ length. All Au beads were forced to move as a rigid body during the simulation time. Each configuration was first relaxed for $1x10^4$ steps with

a time step of $\Delta t = 0.01\tau$. After, at least additional 6x10[6] time steps ($\Delta t = 0.02\tau$) were performed for productive runs. Electrostatic interactions were treated by means of the smooth particle mesh Ewald method. System equilibration was assessed by monitoring temperature, pressure, density, and potential energy profile as well as composition of nearest neighbours of the sulphur beads. Each system was tested on three independently generated starting configurations.

The optimized adimensional values for the bond force $\boldsymbol{f}_{ij}^{bond}$ are the following: k_b(C-C) = 10, r_0(C-C) = 0.60, k_b(C-N) = 10, r_0 (C-N) = 0.60, k_b(N-L) = 10, r_0 (N-L) = 0.60, k_b(C-Q) = 10, r_0 (C-Q) = 0.65, k_b(C-P) = 10, r_0 (C-P) = 0.74, k_b(C-F) = 10, r_0 (C-F) = 0.65, k_b(F-F) = 10, r_0 (F-F) = 0.69.

Bond angle adimensional parameters in $\boldsymbol{f}_{ijz}^{angle}$ are: k_θ(C-C-C) = 10, θ_0(C-C-C) = 120, k_θ(C-C-N) = 10, θ_0(C-C-N) = 110,k_θ(C-N-L) = 10, θ_0(C-N-L) = 109, k_θ(C-C-Q) = 100, θ_0(C-C-Q) = 110, k_θ(C-P-C) = 10, θ_0(C-P-C) = 110, k_θ(P-C-N) = 10, θ_0(P-C-N) = 110, k_θ(C-N-L) = 40, θ_0(C-N-L) = 109, k_θ(L-N-L) = 40, θ_0(L-N-L) = 109, k_θ(C-F-F) = 10, θ_0 (C-F-F) = 110, k_θ(F-F-F) = 10, θ_0 (F-F-F) = 110. Lastly, the DPD nonbonded parameters a_{ij}, are listed in **Table S3**.

Table S3. DPD nonbonded parameters a_{ij} for **NP1-5/6**.

a_{ij}	Au	S	C	P	N	L	F	Q	W/CI
**Au**	49.6								
**S**	1.90	48.3							
**C**	55.4	52.5	50.1						
**P**	70.2	77.2	63.7	62.6					
**N**	75.7	79.8	58.4	55.6	76.6				
**L**	58.1	66.4	52.1	47.1	56.9	51.4			
**F**	58.3	77.6	66.3	63.7	68.9	60.2	56.8		
**Q**	78.3	80.1	68.9	-	-	-	55.1	72.6	
**W/CI**	80.5	78.3	82.8	52.8	42.3	61.1	62.8	36.0	51.6

DPD calculations were performed using the Culgi simulation package (v.12.0, Culgi B.V., Leiden, The Netherlands). The force cutoff radius r_c, the particle mass m_i, and k_BT were taken as units of length, mass and energy.

S3.2. Molecular dynamics (MD)

At atomistic level ligand **1-6** were prepared using antechamber and assigning gaff2[11] atom types; force field parameters for the radical probe **7** were taken from the works of Barone et al.[12-13] Partial charges were calculated applying the RESP method provided by RED server. Au-Au interactions were described with the

parameters of INTERFACE[14] force field for metals. A harmonic bond was created between each sulfur atom and a gold atom within 3.3 Å with a spring constant 50.000 kJ/mol*nm^2.[15] Ligands positions on the Au surface were mapped back from DPD calculations for **NP1-5/6** (**Section S3.1**).

Using tleap[16] program in combination with Packmol, each system was then solvated with TIP3P water, extending at least 20 Å from each solute atom, and counterions were added to neutralize the system. A combination of steepest descent (10000 cycles) and conjugate gradient (10000 cycles), followed by a heating phase of 100 ps in NVT ensemble (integration step = 1 fs), was carried out to reach the production temperature of 300 K (or 340K). Then, density was equilibrated for at least 50 ns in NPT conditions (integration step = 2 fs, pressure 1 atm), while pressure was maintained by Berendsen barostat. Finally, we switched to Monte Carlo barostat implemented in AMBER 18[16] suite of programs for production run, of which the first part is discarded until equilibration of ligands root-mean-square-deviation was reached. Data collection (400 ns) is stored from this point on. Temperature was controlled by Langevin method (damping coefficient of 5 ps^{-1}) throughout all simulations. Electrostatic interactions were computed by means of Particle Mesh Ewald algorithm, and calculations were carried out using AMBER 18. For systems containing the probe, the radical was placed close to the equilibrated monolayer (not in contact) changing initial position and orientation of the probe with respect to the NP and assigning different starting velocities to enhance the sampling of the binding for a total of 1.2 μs time of simulation.

Analysis was mainly performed on production runs via AMBER analysis tools, and by in-house developed Python scripts. Gold size, ligand number and composition were chosen to match those found in the experiments (see **Section S2** and ref.[17]) and are summarized in **Table S4** for convenience of the reader.

Table S4. Summary of gold core size and monolayer composition used to build each molecular model.

	**Core size (nm)**	**Number of ligands**
**NP1**	4.4	385
**NP1/6**	4.5	245/117
**NP2**	4.2	326
**NP2/6**	4.1	237/103
**NP3**	4.1	330
**NP3/6**	4.5	239/132
**NP4**	4.4	384
**NP4/6**	4.5	275/125
**NP5**	4.4	360
**NP5/6**	4.2	244/98

S3.3. Voronoi tessellation and area dispersion index (ADI)

The analysis of distribution of self-assembled ligands was carried out by performing a Voronoi tessellation[18] by using the Python package *scipy*[19] on 400 frames taken from production runs. The Euclidean coordinates (*x*, *y*, *z*) of each ligand center of mass were first projected onto a bi-dimensional (φ, cos (θ)) plane; in this projected plane, a Voronoi tessellation was calculated and the boundary cells of the system were then excluded to avoid irregularities. To quantify the regularity of the computed Voronoi tessellations, a measure of dispersion denominated area dispersion index (ADI) was calculated according to the relation:

$$ADI = \frac{\sigma^2}{\mu}$$

where μ is the mean and σ^2 is the variance for the selected system computed on the borderless Voronoi polygons areas. This measure was calculated on each system, and to ensure commensurability a step of normalization was carried out, in which each Voronoi polygon area is normalized on the total Voronoi tessellation area of the system. Although the index is rather simple and an indication of uniformity in distributions, it should not be used for systems that show a Poisson distribution of the areas, distribution that wasn't present in any of our systems.

S3.4. Smooth Overlap of Atomic Position (SOAP)

The Smooth Overlap of Atomic Position (SOAP)[20-21] is a state-of-art, general-purpose, atom-centered, density-based 3D fingerprint that encodes an atomic region or environment coming from an atomistic simulation. So far, the SOAP formalism has been used with considerable success in material informatics for properties prediction and structural classification, among others.[22-24] The key concept is the representation of the atomic density around an atom *j* as a sum of smeared Gaussians centered on each surrounding atom of species α

$$\rho^a(\boldsymbol{r}) = \sum_{i \in a} f_c(\boldsymbol{r}_{ij})\, g(\boldsymbol{r} - \boldsymbol{r}_{ij})$$

where f_c is a cutoff function going to zero within a cutoff distance r_c, which determines the amplitude of the local environment. The atom density is expanded in terms of a basis of orthogonal radial basis functions $g_n(r)$ and spherical harmonics $Y_{lm}(\theta, \phi)$[25]

$$\rho^a(\boldsymbol{r}) \cong \sum_{a \in r_c} \sum_{nlm} c^{\alpha}_{nlm}\; g_n(r)\, Y_{lm}(\theta, \phi)$$

where c_{nlm}^{α} are the spherical harmonics and radial functions expansion coefficients, *n* is an index related to the radial expansion, and *l*, *m* to the spherical harmonics. The coefficients c_{nlm}^{a} are obtained by the inner product

$$c_{nlm}^{a} = \sum_{a \in r_c} \sum_{nlm} \rho^{a}(r)\ g_n(r)\, Y_{lm}(\theta, \phi) = \iiint_{R^3} dV\ g_n(r)\ \rho^{a}(r)\, Y_{lm}(\theta, \phi)$$

By integration over all relative rotations, we obtain a representation of the atomic environment that is not only invariant from translations and permutations but also spherical invariant.

For multicomponent environments (i.e., containing more than one atomic species), the SOAP partial power spectrum vector takes the form of

$$p_{nn'l}^{a,b} = \pi \sqrt{\frac{8}{2l+1}} \sum_{m} c_{nlm}^{a} * c_{n'lm}^{b}$$

where *n* and *n'* are indices for the different radial basis function, *l* is the angular degree of spherical harmonics and *a* and *b* are the atomic species. The SOAP power spectrum vector (or SOAP vector) is as an explicit, general, and complete representation of chemical environments.

The SOAP vector of the reporter molecule **7** was computed by considering the nitrogen atom as the center of each atomic environment. The no-peripherical position of the nitrogen atom is expected to minimize the noise associated with the intrinsic molecular conformational mobility and to facilitate environments discrimination. The SOAP calculation was carried out by using the *Dscribe*[26] Python package with the following parameters (and by leaving the other parameters to default):

- SOAP medium-range: $l = n = 8$; cutoff $r_1 = 9.0$ Å
- SOAP short-range: $l = n = 8$; cutoff $r_2 = 4.5$ Å.

The cutoff radius restricts the contributions to the density to the atoms within $r_{ji} < r_c$. Higher r_c, higher is the information encoded in the SOAP vectors, but also higher is the computational cost associated with their evaluation or transformation. A reasonable choice for describing local environments in soft matter[27] is to set the cutoff radius just after the first peak of the Radial Distribution Function (RDF) from the SOAP center of the reporter. For our systems, this led to a cutoff $r_1 = 9.0$ Å, and $r_2 = 4.5$ Å, (**Figure S23**).

Due to the large number of chemical species in our systems, the computed SOAP features space included 14354 (considering all atoms) and 4752 (considering only solvent molecules) features for the medium-range and short-range SOAP, respectively. The SOAP analysis was carried out extracting 400 configurations taken from the equilibrated full molecular dynamics trajectory.

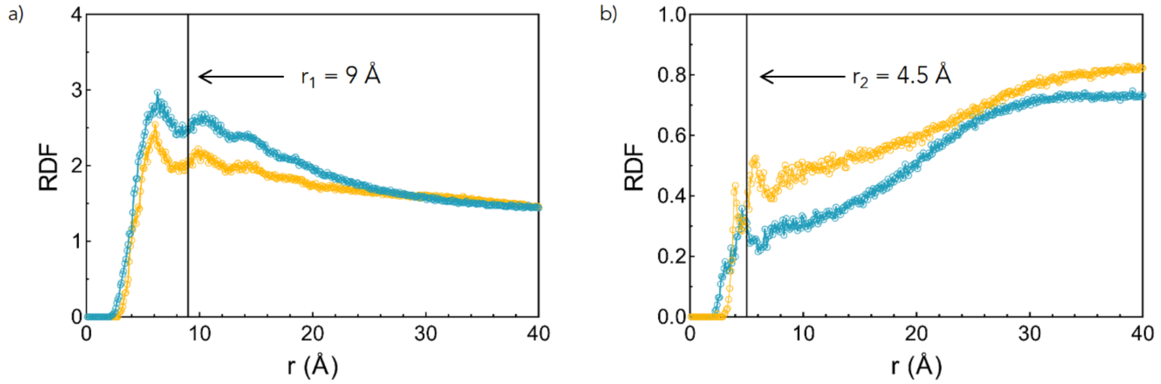

Figure S23. Examples of radial distribution function (RDF) calculated from the nitrogen atom of the probe including all atoms (a) and only oxygens of water molecules (b) in **NP2** (●) and **NP4** (●). The line shows the cutoff chosen for (a) SOAP-medium range, namely r_1, and (b) SOAP-short range, namely r_2.

To increase the computational efficiency of the SOAP analysis and to obtain a low-dimensional representation to simplify the interpretation of the results, a linear Principal Component Analysis (PCA) was carried out with the *scikit-learn*[28] Python package. This algorithm linearly transforms a set of correlated data into a new set of values linearly uncorrelated denominated principal components. We thus reduced each high dimensional SOAP features to the first 10 principal components for the medium-range SOAP vectors, and to the first 10 principal components merging all the short-range SOAP vectors, maintaining a variance of at least 94% in both cases (**Figure S24**).

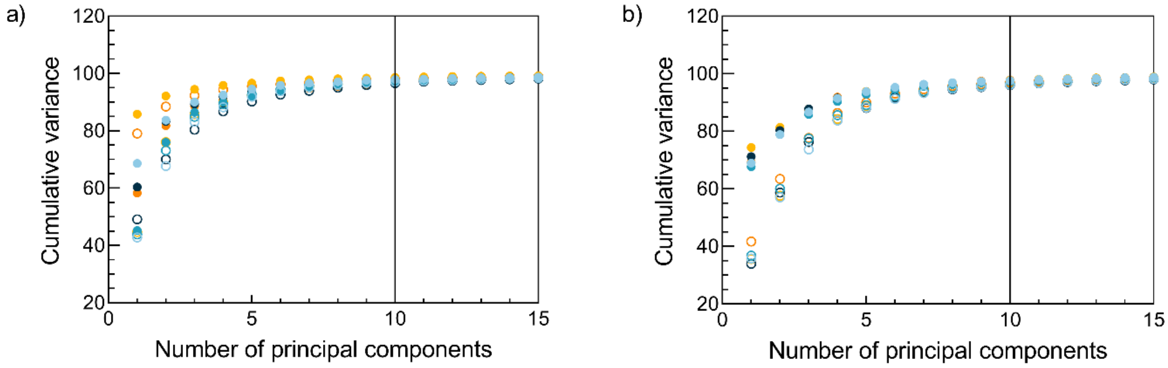

Figure S24. Cumulative variance as a function of the number of principal components for medium-range (a) and short-range (b) SOAP vectors. Color legend: ● NP1, ○ NP1/6, ● NP2, ○ NP2/6, ● NP3, ○ NP3/6, ● NP4, ○ NP4/6, ● NP5, ○ NP5/6.

S3.5. Gaussian mixtures clustering algorithm

The clustering of the local environments was performed by the Gaussian mixtures model (GMM), implemented in the *scikit-learn*[28] Python package. GMM is a distribution-based clustering algorithm, which

parametrically fits the probability distribution of a set of points **x** to a sum of N multidimensional Gaussians

$$p(\boldsymbol{x}) = \sum_{i=1}^{N} \phi_i\, \mathcal{N}(x|\mu_i, \Sigma_i)$$

where ϕ_i is the fraction of the probability belonging to the Gaussian $\mathcal{N}$ (i. e., the relative weight of the cluster *i*-th), μ_i is its mean and Σ_i its covariance

$$\mathcal{N}(x|\mu_i, \Sigma_i) = \frac{1}{\sqrt{(2\pi)^N|\Sigma_i|}}\, exp(-\frac{(x-\mu_i)^T\Sigma_i^T(x-\mu_i)}{2})$$

The parameters ϕ_i, μ_i, and Σ_i of the model are assigned by the expectation-maximization algorithm (EM),[29] an iterative method for finding maximum likelihood ($\mathcal{L}$) estimates of parameters in statistical models, which for identically independently distributed data takes the form of

$$\mathcal{L} = \ln p(x \mid \phi, \mu, \Sigma) = \sum_{k=1}^{K} \ln\,(\sum_{i=1}^{N} \phi_i\, \mathcal{N}(x_k|\mu_i, \Sigma_i))$$

The automatic identification of the number N of clusters (i.e. local environments) for each system is carried out by minimization of the Bayesian Information Criteria (BIC),[30] which is a complex penalty added to the log-likelihood in the form

$$BIC = k \ln(n) - 2\mathcal{L}$$

where k is the number of parameters, including N, estimated by the model and n is the number of data.

Every Gaussian mixture uses a full covariance and the BIC minimization is performed on each system separately. Consistency (or quality) of the clusterization is then assessed by calculating silhouettes scores (SC), using the implementation available in *scikit-learn* package. Overall, no negative SC values have been found, thus indicating proper cluster assignment: **NP2** 0.23, **NP4** 0.42, **NP5** 0.27, **NP1/6** 0.27, **NP2/6** 0.33, **NP3/6** 0.41, **NP4/6** 0.44, **NP5/6** 0.60. In line with MD calculations and ESR measurements, higher SC coefficients are associated to local environments with well distinct features (e.g., in terms of hydration, local hydrophobicity, a_N) that allow stronger cluster attribution, while lower SC values to systems where differences between the environments are smoother, thus hindering the separation of well-defined clusters and making the attribution of the microstate to the exact cluster less certain.

The GMM probabilistic framework was selected against other types of performing clustering algorithms (e.g., HDBSCAN, hierarchical k-means, Probabilistic Analysis of Molecular Motifs (PAMM)) because of the high dimensionality of the data set to treat. Due to the large dimension of the features space, the Euclidean distance between each SOAP vectors could penalize the density based clustering techniques and bias the result

towards microclusters or classify possible data as noise. While this could be avoided by carefully selecting the clustering parameters (HDBSCAN, hierarchical k-means) or by choosing a dendrogram cutoff (PAMM), the GMM paired with the EM algorithm and BIC minimization is able to identify macroclusters with the minimum human intervention. Constraint to the adoption of GMM in the clusterization step is the convexity of the clusters to be identified.

S3.6. Similarity measure

To compare two different atomic local environments (i,j) defined by their respective SOAP vectors, a similarity measure defined from the inner product between the two atom densities was adopted. Integrated over all possible three-dimensional rotations $\tilde{R}$ it leads to a SOAP similarity linear kernel[20]

$$\tilde{K}(i,j) = \int d\tilde{R} \left| \iint \rho(r)^i \rho(\hat{R}r)^j dr \right|^n$$

and from setting $n = 2$ to retain the angular information of the original environments and with some algebraic reformulation the kernel can be expressed as

$$\tilde{K}(i,j) = \tilde{\boldsymbol{p}}_i \cdot \tilde{\boldsymbol{p}}_j$$

where $\tilde{\boldsymbol{p}}_i$ is the non-normalized SOAP power spectra of the environment i.

The kernel is then normalized to enable the direct comparison between the different SOAP vectors in the form of a normalized SOAP linear kernel product:

$$K(i,j)^{SOAP} = \frac{\tilde{K}(i,j)}{\sqrt{\tilde{K}(i,i)\tilde{K}(j,j)}} = \frac{\tilde{\boldsymbol{p}}_i \cdot \tilde{\boldsymbol{p}}_j}{\sqrt{(\tilde{\boldsymbol{p}}_i \cdot \tilde{\boldsymbol{p}}_i)(\tilde{\boldsymbol{p}}_j \cdot \tilde{\boldsymbol{p}}_j)}} = \boldsymbol{p}_i \cdot \boldsymbol{p}_j$$

in which $\boldsymbol{p}_i$ is the normalized SOAP power spectra of the environment i.

The similarity obtained from the normalized SOAP linear kernel ranges between 0 and 1 and explains how similar a system is to another: 0 is completely different (systems are not superimposed) and 1 if the systems are equals (systems are superimposed). After the computation of the similarity score, the distance between two points (i.e. environments) in a Euclidean n-space can be calculated as[21]

$$d_{SOAP}(i,j) = \sqrt{K(i,i) + K(j,j) - 2K(i,j)} = \sqrt{2 - 2\,K(i,j)} = \sqrt{2 - 2\,\boldsymbol{p}_i \cdot \boldsymbol{p}_j}$$

The distances between environments were then displayed in a distance matrix, where they were sorted by accounting of every other environment distance, thus finally leading to the similarity matrix reported in **Figure 7** and **S11**.

S4. ELECTRON SPIN RESONANCE (ESR)

Monolayer features can be investigated by molecular probes, which are able both to enter inside the monolayer and to possess spectral features that depends on the molecular environment of the surroundings. Functionalized benzyl *tert*-bytulnitroxides (BTBN), possess such characteristics and have been largely employed to characterized different type of water-soluble protected gold nanoparticles.[31-34] In the presence of water-soluble protected gold nanoparticles, when a nitroxide probe is located in the organic compartment of the monolayer, isotropic nitrogen hyperfine splitting constant, a_N, is significantly smaller than that measured in water and it is possible to distinguish different EPR signals for the two different environments and thus to measure the partition equilibrium constant of the organic probe which is strictly related to the monolayer composition. The responsiveness of a_N to the nature of the environment surrounding the nitroxide probe is a consequence of the resonance hybrid formulation of nitroxidic functionality in which three π electrons are distributed over the nitrogen and oxygen atomic centers. In particular, the greater the polarity of the medium surrounding the radical center, the more important is the dipolar structure (Structure B, **Scheme S1**) having both a larger spin density on the nitrogen and thus a larger value of the nitrogen hyperfine coupling.

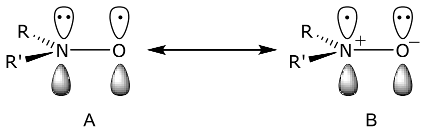

Scheme S1. Resonance structures of nitroxides.

S5. SYNCHROTRON-BASED X-RAY PHOTOELECTRON SPECTROSCOPY (XPS)

In **Figure S25**, we report a typical series of Au 4f, C 1s and S 2p spectra, acquired on the **NP2** sample, which is representative of each of the samples we have measured. The Au 4f and C 1s core level were acquired with a photon energy of hv = 410 eV, while the S 2p was acquired with a photon energy of 360 eV. The BE scale was aligned with the main component of the C 1s, which is the tabled C=C bond of the thiols (BE = 284.9). It was possible to identify the Au 4f spin-orbit doublet (spin-orbit splitting ΔE = 3.7 eV), with the Au $4f_{7/2}$ component found at BE = 83.9 eV, a value which is compatible with the presence of Au(0).[33] Similarly, the S 2p shows a spin-orbit doublet located at BE=162.2 eV for S $2p_{3/2}$ (spin-orbit splitting ΔE = 1.2 eV), consistent with earlier reports.[33] The determination of the organic layer thickness was carried out by extrapolating the intensity of the photoemission signal of C 1s and Au 4f spectra and applying the procedure described by Shard for nanoscopic particles, whose mean core radius is known.[2]

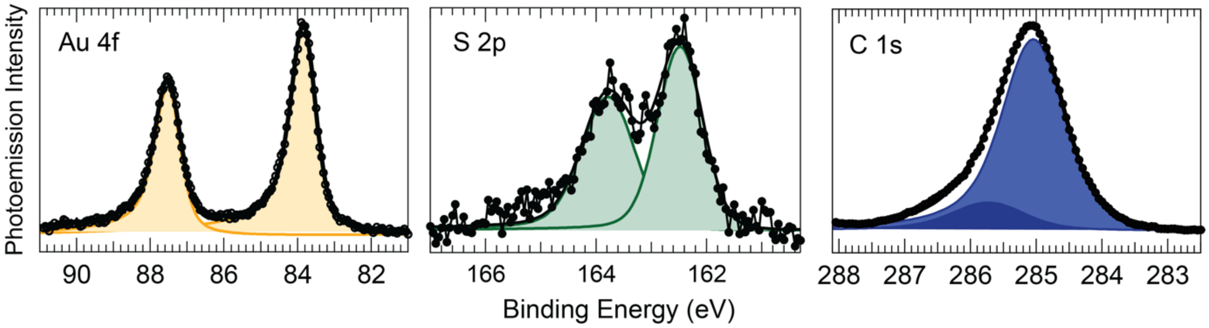

Figure S25. Background-subtracted Au4f, S 2p and C 1s core level spectra acquired for the **NP2** sample. The black dots are the experimental data, the black continuous line is the best fit to the spectrum obtained using the spectral components (indicated in color).

S6. REFERENCES

1. Campello, R. J. G. B.; Moulavi, D.; Sander, J. In *Density-based clustering based on hierarchical density estimates*, Advances in Knowledge Discovery and Data Mining, Berlin, Heidelberg, 2013; Pei, J.; Tseng, V. S.; Cao, L.; Motoda, H.; Xu, G., Eds. Springer Berlin Heidelberg: Berlin, Heidelberg, 2013; pp 160-172.
2. Shard, A. G. A straightforward method for interpreting XPS data from core–shell nanoparticles. *J. Phys. Chem. C* **2012**, *116*, 16806-16813.
3. Español, P.; Warren, P. B. Perspective: Dissipative particle dynamics. *J. Chem. Phys.* **2017**, *146*, 150901.
4. Beránek, P.; Posocco, P.; Posel, Z. Phase behavior of gradient copolymer melts with different gradient strengths revealed by mesoscale simulations. *Polymers* **2020**, *12*, 2462.
5. Bray, D. J.; Anderson, R. L.; Warren, P. B.; Lewtas, K. Wax formation in linear and branched alkanes with dissipative particle dynamics. *J. Chem. Theory* **2020**, *16*, 7109-7122.
6. Zhang, F.; Peng, S.; Xu, J.; Cai, C.; Zhang, L. Morphological transitions of micelles induced by the block arrangements of copolymer blocks: Dissipative particle dynamics simulation. *Phys. Chem. Chem. Phys.* **2022**, *24*, 10757-10764.
7. Posocco, P.; Hassan, Y. M.; Barandiaran, I.; Kortaberria, G.; Pricl, S., et al. Combined mesoscale/experimental study of selective placement of magnetic nanoparticles in diblock copolymer films via solvent vapor annealing. *J. Phys. Chem. C* **2016**, *120*, 7403-7411.
8. Rodrigo, A. C.; Bromfield, S. M.; Laurini, E.; Posocco, P.; Pricl, S., et al. Morphological control of self-assembled multivalent (SAMUL) heparin binding in highly competitive media. *Chem. Commun.* **2017**, *53*, 6335-6338.
9. Posel, Z.; Posocco, P.; Fermeglia, M.; Lísal, M.; Pricl, S. Modeling hierarchically structured nanoparticle/diblock copolymer systems. *Soft Matter* **2013**, *9*, 2936-2946.
10. Martínez, L.; Andrade, R.; Birgin, E. G.; Martínez, J. M. Packmol: A package for building initial configurations for molecular dynamics simulations. *J. Comput. Chem.* **2009**, *30*, 2157-2164.
11. He, X.; Man, V. H.; Yang, W.; Lee, T.-S.; Wang, J. A fast and high-quality charge model for the next generation general AMBER force field. *J. Chem. Phys.* **2020**, *153*, 114502.
12. Barone, V.; Bencini, A.; Cossi, M.; Di Matteo, A.; Mattesini, M., et al. Assessment of a combined QM/MM approach for the study of large nitroxide systems *in vacuo* and in condensed phases. *J. Am. Chem. Soc.* **1998**, *120*, 7069-7078.
13. Improta, R.; di Matteo, A.; Barone, V. Effective modeling of intrinsic and environmental effects on the structure and electron plaramagnetic resonance parameters of nitroxides by an integrated quantum mechanical/molecular mechanics/polarizable continuum model approach. *Theor. Chem. Acc.* **2000**, *104*, 273-279.
14. Heinz, H.; Lin, T.-J.; Kishore Mishra, R.; Emami, F. S. Thermodynamically consistent force fields for the assembly of inorganic, organic, and biological nanostructures: The INTERFACE force field. *Langmuir* **2013**, *29*, 1754–1765.
15. Chew, A. K.; Van Lehn, R. C. Effect of core morphology on the structural asymmetry of alkanethiol monolayer-protected gold nanoparticles. *J. Phys. Chem. C* **2018**, *122*, 26288-26297.
16. D. A. Case, I. Y. Ben-Shalom, S. R. Brozell, D. S. Cerutti, I. T. E. Cheatham, V. W. D. Cruzeiro, T. A. Darden, R. E. Duke, D. Ghoreishi, M. K. Gilson, H. Gohlke, A. W. Goetz, D. Greene, R. Harris, N. Homeyer, S. Izadi, A. Kovalenko, T. Kurtzman, T. S. Lee, S. LeGrand, P. Li, C. Lin, J. Liu, T. Luchko, R. Luo, D. J. Mermelstein, K. M. Merz, Y. Miao, G. Monard, C. Nguyen, H. Nguyen, I. Omelyan, A. Onufriev, F. Pan, R. Qi, D. R. Roe, A. Roitberg, C. Sagui, S. Schott-Verdugo, J. Shen, C. L. Simmerling, J. Smith, R. Salomon-Ferrer, J. Swails, R. C. Walker, J. Wang, H. Wei, R. M. Wolf, X. Wu, L. Xiao, D. M. York and P. A. Kollman, AMBER 2018, University of California, San Francisco.
17. Pellizzoni, E.; Şologan, M.; Daka, M.; Pengo, P.; Marson, D., et al. Thiolate end-group regulates ligand arrangement, hydration and affinity for small compounds in monolayer-protected gold nanoparticles. *J. Colloid Interface Sci.* **2022**, *607*, 1373-1381.
18. Bock, M.; Tyagi, A. K.; Kreft, J.-U.; Alt, W. Generalized Voronoi tessellation as a model of two-dimensional cell tissue dynamics. *Bull. Math. Biol.* **2010**, *72*, 1696-1731.

19. Virtanen, P.; Gommers, R.; Oliphant, T. E.; Haberland, M.; Reddy, T., et al. Scipy 1.0: Fundamental algorithms for scientific computing in Python. *Nat. Methods* **2020**, *17*, 261-272.
20. Bartók, A. P.; Kondor, R.; Csányi, G. On representing chemical environments. *Phys. Rev. B* **2013**, *87*, 184115.
21. De, S.; Bartók, A. P.; Csányi, G.; Ceriotti, M. Comparing molecules and solids across structural and alchemical space. *Phys. Chem. Chem. Phys.* **2016**, *18*, 13754-13769.
22. Bernstein, N.; Bhattarai, B.; Csányi, G.; Drabold, D. A.; Elliott, S. R., et al. Quantifying chemical structure and machine-learned atomic energies in amorphous and liquid silicon. *Angew. Chem. Int. Ed.* **2019**, *58*, 7057-7061.
23. Musil, F.; De, S.; Yang, J.; Campbell, J. E.; Day, G. M., et al. Machine learning for the structure–energy–property landscapes of molecular crystals. *Chem. Sci.* **2018**, *9*, 1289-1300.
24. Paruzzo, F. M.; Hofstetter, A.; Musil, F.; De, S.; Ceriotti, M., et al. Chemical shifts in molecular solids by machine learning. *Nat. Commun.* **2018**, *9*, 4501.
25. Musil, F.; Veit, M.; Goscinski, A.; Fraux, G.; Willatt, M. J., et al. Efficient implementation of atom-density representations. *J. Chem. Phys.* **2021**, *154*, 114109.
26. Himanen, L.; Jäger, M. O. J.; Morooka, E. V.; Federici Canova, F.; Ranawat, Y. S., et al. Dscribe: Library of descriptors for machine learning in materials science. *Comput. Phys. Commun.* **2020**, *247*, 106949.
27. Gasparotto, P.; Bochicchio, D.; Ceriotti, M.; Pavan, G. M. Identifying and tracking defects in dynamic supramolecular polymers. *J. Phys. Chem. B* **2020**, *124*, 589-599.
28. Pedregosa, F.; Varoquaux, G.; Gramfort, A.; Michel, V.; Thirion, B., et al. Scikit-learn: Machine learning in Python. *J. Mach. Learn. Res.* **2011**, *12*, 2825–2830.
29. Dempster, A. P.; Laird, N. M.; Rubin, D. B. Maximum likelihood from incomplete data via the EM algorithm. *J. Roy. Stat. Soc. Ser. B. (Stat. Method.)* **1977**, *39*, 1-38.
30. Schwarz, G. Estimating the dimension of a model. *Ann. Stat.* **1978**, *6*, 461-464.
31. Ionita, P.; Caragheorgheopol, A.; Gilbert, B. C.; Chechik, V. EPR study of a place-exchange reaction on Au nanoparticles: Two branches of a disulfide molecule do not adsorb adjacent to each other. *J. Am. Chem. Soc.* **2002**, *124*, 9048-9049.
32. Lucarini, M.; Franchi, P.; Pedulli, G. F.; Gentilini, C.; Polizzi, S., et al. Effect of core size on the partition of organic solutes in the monolayer of water-soluble nanoparticles: An ESR investigation. *J. Am. Chem. Soc.* **2005**, *127*, 16384-16385.
33. Gentilini, C.; Evangelista, F.; Rudolf, P.; Franchi, P.; Lucarini, M., et al. Water-soluble gold nanoparticles protected by fluorinated amphiphilic thiolates. *J. Am. Chem. Soc.* **2008**, *130*, 15678-15682.
34. Gentilini, C.; Franchi, P.; Mileo, E.; Polizzi, S.; Lucarini, M., et al. Formation of patches on 3D SAMs driven by thiols with immiscible chains observed by ESR spectroscopy. *Angew. Chem. Int. Ed.* **2009**, *48*, 3060-3064.
